# Risk factors for severe reactions in food allergy: Rapid evidence review with meta‐analysis

**DOI:** 10.1111/all.15318

**Published:** 2022-04-28

**Authors:** Paul J. Turner, Stefania Arasi, Barbara Ballmer‐Weber, Alessia Baseggio Conrado, Antoine Deschildre, Jennifer Gerdts, Susanne Halken, Antonella Muraro, Nandinee Patel, Ronald Van Ree, Debra de Silva, Margitta Worm, Torsten Zuberbier, Graham Roberts, the Global Allergy, Asthma European Network (GA2LEN) Food Allergy Guideline Group

**Affiliations:** ^1^ National Heart & Lung Institute Imperial College London London UK; ^2^ Translational Research in Paediatric Specialities Area Division of Allergy Bambino Gesù Children's Hospital IRCCS Rome Italy; ^3^ Clinic for Dermatology and Allergology Kantonsspital St. Gallen St. Gallen Switzerland; ^4^ Department of Dermatology University Hospital Zürich Zürich Switzerland; ^5^ CHU Lille, Univ. Lille Pediatric Pulmonology and Allergy Department Hôpital Jeanne de Flandre Lille France; ^6^ Food Allergy Canada Toronto Ontario Canada; ^7^ Hans Christian Andersen Children’s Hospital Odense University Hospital Odense Denmark; ^8^ Food Allergy Centre Padua General University Hospital Padua Italy; ^9^ Departments of Experimental Immunology and of Otorhinolaryngology Amsterdam University Medical Centers, location AMC Amsterdam The Netherlands; ^10^ The Evidence Centre London UK; ^11^ Division of Allergy and Immunology Department of Dermatology, Venerology and Allergy Charité, Universitätsmedizin Berlin Berlin Germany; ^12^ NIHR Southampton Biomedical Research Centre University Hospital Southampton NHS Foundation Trust Faculty of Medicine University of Southampton Southampton UK; ^13^ The David Hide Asthma and Allergy Research Centre St Mary's Hospital Isle of Wight UK

**Keywords:** anaphylaxis, biomarkers, food allergy, risk assessment, severity

## Abstract

This rapid review summarizes the most up to date evidence about the risk factors for severe food‐induced allergic reactions. We searched three bibliographic databases for studies published between January 2010 and August 2021. We included 88 studies and synthesized the evidence narratively, undertaking meta‐analysis where appropriate. Significant uncertainties remain with respect to the prediction of severe reactions, both anaphylaxis and/or severe anaphylaxis refractory to treatment. Prior anaphylaxis, an asthma diagnosis, IgE sensitization or basophil activation tests are not good predictors. Some molecular allergology markers may be helpful. Hospital presentations for anaphylaxis are highest in young children, yet this age group appears at lower risk of severe outcomes. Risk of severe outcomes is greatest in adolescence and young adulthood, but the contribution of risk taking behaviour in contributing to severe outcomes is unclear. Evidence for an impact of cofactors on severity is lacking, although food‐dependent exercise‐induced anaphylaxis may be an exception. Some medications such as beta‐blockers or ACE inhibitors may increase severity, but appear less important than age as a factor in life‐threatening reactions. The relationship between dose of exposure and severity is unclear. Delays in symptom recognition and anaphylaxis treatment have been associated with more severe outcomes. An absence of prior anaphylaxis does not exclude its future risk.

## 1 INTRODUCTION

Estimating the risk of severe reactions is one of the most significant knowledge gaps in managing people with food allergy. Near‐fatal and fatal anaphylaxis to food are rare: the estimated incidence of fatal food‐anaphylaxis is 1.81 (95% CI 0.94–3.45) per million person‐years.^1^ Near‐fatal anaphylaxis to food (requiring intensive care support) is around 10 times more common, but still rare.^2^ However, such severe reactions are unpredictable.^3^ Most people who die from fatal food‐anaphylaxis only have a history of previous mild reactions.^3^ Our inability to identify those at greater risk of severe reactions means that people with food allergy are often managed as being at equal risk of fatal reactions. This can cause unnecessary anxiety, excessive dietary restriction and reduced health‐related quality of life.

Ideally, clinicians would be able to risk‐stratify people with food allergy, to provide cost‐effective, targeted support to those at greatest risk. Various models incorporating potential risk factors have been proposed—both for *any* anaphylaxis, and anaphylaxis refractory to treatment (hereafter described as *refractory* anaphylaxis)^4^, ^5^; however, the underlying evidence is disparate. Some useful narrative reviews have begun to draw the evidence together,^3^ but we identified no systematic reviews exploring this topic. In this rapid review, we summarize what is known about the risk factors for more severe reactions in people with food allergy.

## 2 METHODS

We systematically searched three bibliographic databases for studies relating to IgE‐mediated food allergy or Food protein induced enterocolitis syndrome (FPIES), published between 1 January, 2010, and 31 August, 2021. We searched from 2010 because other reviews covered earlier literature.^3^ We extracted data in duplicate, assessed the risk of bias and undertook meta‐analysis where appropriate. The online supplement describes the methods in more detail and eligible studies (Tables S1–S5). We included 88 studies (4 systematic reviews, 9 randomized controlled trials and 75 observational studies) and synthesized the evidence according to the categories in Table 1, with an emphasis on modifiable risk factors which could be used to reduce risk.

**TABLE 1:** Potential risk factors for more severe reactions in food allergy

		Burden of allergic disease	Host immune response	Allergen presentation	Host behaviours	Concomitant medications	Non‐modifiable host factors	Management of allergic reaction
Increased risk	High certainty						Age: Adolescence/adults <40 years (food triggers) Older age associated with higher risk of more severe anaphylaxis to non‐food triggers	
	Low certainty	Prior anaphylaxis is not a good predictor of future anaphylaxis Absence of prior anaphylaxis does not exclude future risk	Greater IgE binding (avidity/affinity) Increased effector cell (basophil/mast cell) activation *in vivo* LTP sensitization without pollen co‐sensitization	Specific food triggers e.g. persisting cow's milk allergy, peanut, seafood, wheat Potential impact of food matrix	Risk‐taking behaviour Situational awareness Exercise	ACE inhibitors Beta‐blockers	Sex (males) Older age (food triggers only)	Delays in treatment
Decreased risk	Low certainty			Specific food triggers e.g. egg		Disease‐modifying treatments e.g. allergen immunotherapy		
	High certainty		Bet v 1‐mediated pollen food allergy syndrome					
Unlikely to be a useful predictor in clincial practice		Prior anaphylaxis Well‐controlled asthma	IgE sensitization (skin prick test or blood test) to whole allergens		Risk‐taking behaviour		Sex Age	
Risk unknown		Active atopic disease (allergic rhinitis, eczema) Suboptimal asthma control, asthma severity	IgE sensitization to specific components/basophil activation test Mastocytosis Immune activation for example, viral infections	Dose of allergen	Alcohol	NSAIDs	Cardiovascular disease Ability to compensate Genetics	

Note

For the purposes of this table, ‘high certainty’ means the evidence gives confidence in the conclusion that a variable is or is not a risk factor. Low certainty means that there was some evidence that a variable may be a risk factor, but we were not certain of this conclusion or the size of the impact.

### 2.1 Prior history of anaphylaxis

#### 2.1.1 Key finding

Thirteen studies evaluated whether a history of prior anaphylaxis predicted future risk of *any* anaphylaxis. Prior anaphylaxis was not a good predictor, perhaps because severity depends on a range of factors including level of allergen exposure and the presence/absence of co‐factors. For fatal food‐anaphylaxis, most cases are not associated with a history of prior severe reactions.

#### 2.1.2 Evidence

There was no evidence that prior anaphylaxis predicted the risk of fatal or near‐fatal reaction.^3^, ^6^ In the largest reported series of fatal anaphylaxis, over half of food‐related fatalities were in individuals with only prior mild reactions.^7^ In a unique but small study where food challenges were *not* terminated at onset of objective symptoms, 21/27 (78%) peanut‐allergic children had anaphylaxis when given a sufficient dose of allergen. Thus, the absence of prior anaphylaxis (at least in peanut‐allergic children) may reflect insufficient allergen exposure rather than an inherently lower risk of anaphylaxis.^5^, ^8^, ^9^ However, many patients with prior anaphylaxis to a food only experience milder symptoms subsequently, whether due to accidental allergen exposure^6^, ^10^, ^11^, ^12^ or at formal food challenges.^13^, ^14^, ^15^, ^16^, ^17^, ^18^, ^19^, ^20^

### 2.2 Impact of other allergic diseases

#### 2.2.1 Key finding

**FIGURE 1:**
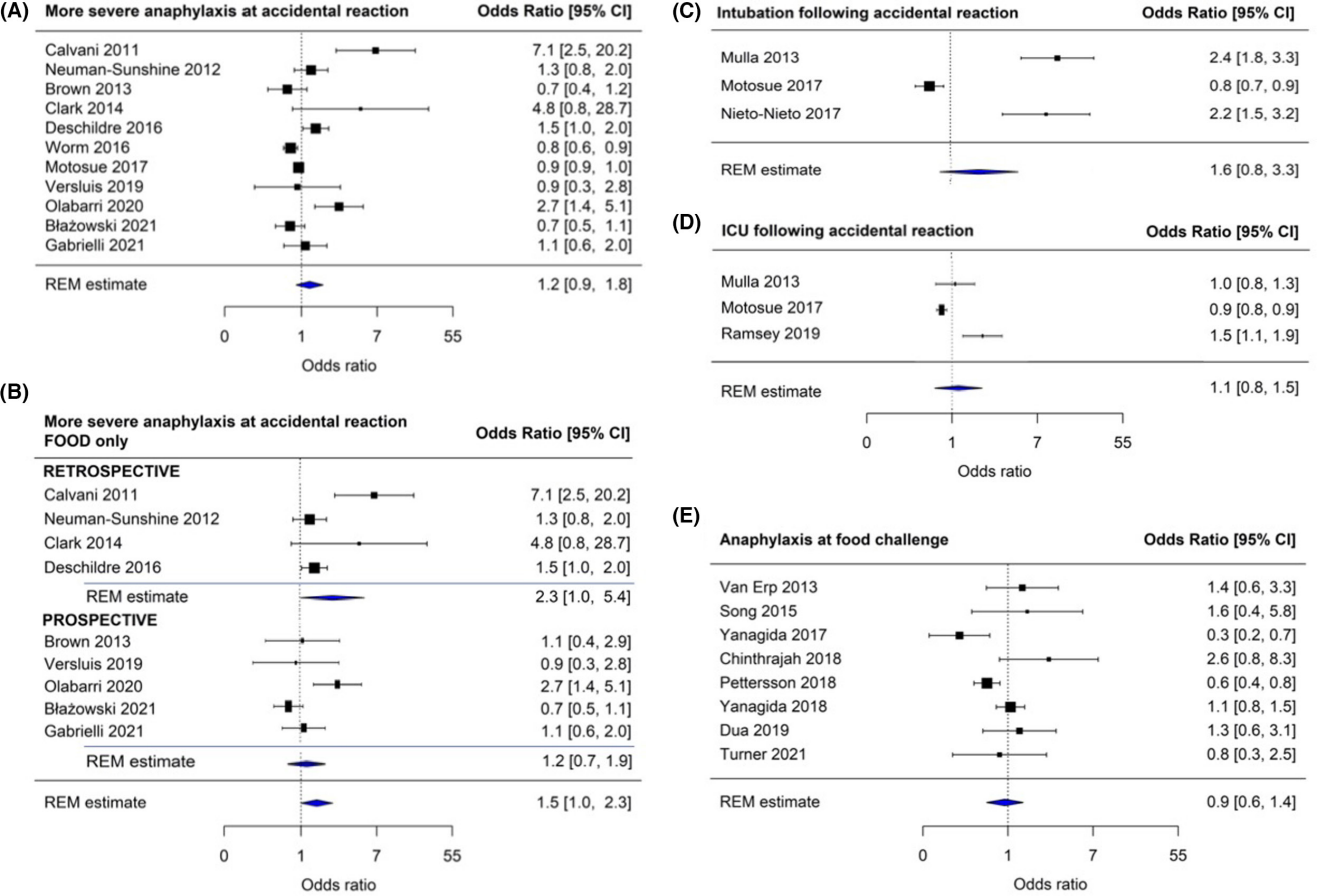
Meta‐analysis of studies reporting impact of asthma on severity of food‐induced allergic reactions. All studies reporting food triggers (A), studies limited to food‐triggered reactions only (B), studies reporting intubation (C) or admission to intensive care (D) as the severity outcome and studies evaluating the impact of asthma on occurrence of anaphylaxis at food challenge (E). The area of each square is proportional to the sample size of the study. CI, confidence interval. Heterogeneity (*I* ^2^ values) are reported in Table 2

Thirty‐four studies (including a systematic review) evaluated the impact of concomitant atopic disease on severity. While diagnosis of asthma was not a risk factor for more severe reactions (Figure 1), it is unclear whether poor asthma control is. In some individuals, active allergic disease of any type may exacerbate severity, but there are no data to suggest an increased risk of fatal or near‐fatal outcomes.

#### 2.2.2 Evidence

Case series of fatal/near‐fatal food‐anaphylaxis indicate that asthma is a common co‐morbidity.^21^, ^22^, ^23^, ^24^ Asthma has therefore been assumed to be a risk factor—something not unreasonable given that the main mechanism of severe outcomes in food allergy involve respiratory compromise.^3^ However, asthma is common, present in over 50% of food‐allergic individuals.^13^, ^25^, ^26^, ^27^, ^28^ As a result, and because fatal reactions are rare,^1^ the value of asthma as a predictor for severe reactions is low. The vast majority (>99.9%) of food‐allergic individuals with asthma will never experience a truly life‐threatening reaction.^3^

We identified 32 primary research studies and one systematic review evaluating the relationship between asthma and severity (Table S6).^10^, ^13^, ^14^, ^15^, ^16^, ^17^, ^18^, ^27^, ^28^, ^29^, ^30^, ^31^, ^32^, ^33^, ^34^, ^35^, ^36^, ^37^, ^38^, ^39^, ^40^, ^41^, ^42^, ^43^, ^44^, ^45^, ^46^, ^47^, ^48^, ^49^, ^50^, ^51^, ^52^ Evidence for asthma as a risk factor was contradictory, even within the same dataset when severity is assessed using different criteria.^41^, ^42^ To address this heterogeneity, we performed a meta‐analysis using a random effects model. We found no consistent evidence that asthma is associated with increased severity of food‐induced reactions (i.e. *any* anaphylaxis) following accidental allergen exposure, or the need for ICU admission and/or intubation and mechanical ventilation (Figure 1 and Table 2). Similarly, there was no association between asthma diagnosis and occurrence of *any* anaphylaxis at food challenge, although challenges are not usually performed in patients with poorly‐controlled asthma. Only in retrospective observational studies, where the risk of bias is greater (Tables S4andS6), was there a weak association between asthma and severity.

**TABLE 2:** Meta‐analysis of studies reporting impact of asthma on severity of food‐induced allergic reactions (Forest plots shown in Figure 1)

Outcome	Number of studies	Pooled OR (95% CI)	Heterogeneity *I* ^2^ (*p* value)
More severe anaphylaxis at accidental reaction	11 studies^10^, ^27^, ^28^, ^31^, ^33^, ^37^, ^41^, ^45^, ^46^, ^48^, ^51^ (trigger = food ± other causes)	1.24 (0.87–1.77)	93% (*p* < .001)
	Limited to studies with unadjusted OR only (8 studies)^10^, ^27^, ^28^, ^31^, ^33^, ^37^, ^45^, ^51^	1.07 (0.79–1.46)	76% (*p* < .001)
	Limited to trigger = food: Retrospective only (4 studies)^10^, ^27^, ^28^, ^33^ Prospective only (5 studies)^31^, ^45^, ^46^, ^48^, ^51^ Combined (9 studies)	2.34 (1.02–5.36) 1.16 (0.71–1.92) 1.52 (0.99–2.31)	75% (*p* < .001)
Intubation following accidental reaction	3 studies^30^, ^36^, ^37^	1.64 (0.82–3.25)	95% (*p* < .001)
ICU admission following accidental reaction	3 studies^30^, ^37^, ^44^	1.08 (0.81–1.45)	87% (*p* = .003)
More severe anaphylaxis at food challenge	8 studies^13^, ^14^, ^15^, ^17^, ^18^, ^35^, ^38^, ^40^	0.93 (0.61–1.43)	70% (*p* = .006)

Most studies, however, do not distinguish between a diagnosis of asthma and the degree of asthma control/underlying airway inflammation. Although evidence is lacking, asthma control may be more relevant than an asthma diagnosis *per se*. In the UK Fatal Anaphylaxis Registry, over 50% of cases had no evidence of asthma exacerbation in the weeks preceding the fatal event.^7^ A retrospective study reported that peanut/tree nut‐allergic patients with prior hospital admission for asthma were more likely to experience bronchospasm at historical reactions (OR 6.8, 95% CI 4.1–11.3), but was not included in this analysis due to publication prior to 2008.^53^ This study is unfortunately affected by methodological inconsistencies and high risk of bias, but remains the only report identified which has attempted a more discriminatory approach to asthma control. It was included in a 2021 meta‐analysis which—in contrast to our meta‐analysis—did find a weak association between asthma and severity (OR 1.89, 95% CI 1.26–2.83).^52^ This might be due to the inclusion of studies at medium‐high risk of bias in the 2021 meta‐analysis, and also that only two of the 13 studies included were specific to food.

Eleven papers evaluated the impact of a diagnosis of eczema and/or allergic rhinitis on severity of food‐induced reactions. Most reported no association with eczema^10^, ^15^, ^16^, ^17^, ^29^, ^37^, ^51^, ^54^ or allergic rhinitis.^10^, ^15^, ^16^, ^29^, ^33^, ^54^ In a prospective multicentre study of anaphylaxis presenting to Emergency Departments in Canada, severe reactions to fruit were more likely to occur in the springtime (OR 1.12, 95% CI 1.03–1.23, adjusted for age, sex, asthma and pre‐hospital treatment).^55^ There was a weak association between eczema and severity (adjusted OR 1.17, 95% CI 1.03–1.34). In contrast, a prospective study of food‐allergic adults in The Netherlands found no impact of active rhinitis on reaction severity.^45^ In the United Kingdom, hospital admissions for food‐induced anaphylaxis (but not fatal food‐anaphylaxis) appear to be more common in the pollen season.^3^, ^56^ However, this has not been observed for paediatric admissions to intensive care due to anaphylaxis in North America.^44^ This may be because the baseline risk of fatal and near‐fatal reactions is so small^1^, ^2^ that any impact of concomitant active atopic disease is negligible.

### 2.3 Host immune response: IgE‐sensitization

#### 2.3.1 Key findings

Twenty‐five  studies examined IgE‐sensitization and/or basophil activation tests. Current evidence is that these do not adequately predict severity, although many studies report a correlation. For some foods, molecular allergology may be useful in predicting higher or lower risk of anaphylaxis, particularly when combined with other potential predictors. For tree nuts, IgE against 2S albumins has been reported to be associated with increased rate of *any* anaphylaxis.^49^, ^51^, ^57^ For peanut, IgE‐monosensitization to Ara h 8 is associated with a lower risk and usually implies pollen food allergy syndrome (PFAS).^58^ LTP‐sensitization in the absence of co‐sensitization to pollens may imply higher risk in some regions.^59^, ^60^

#### 2.3.2 IgE‐sensitization

**FIGURE 2:**
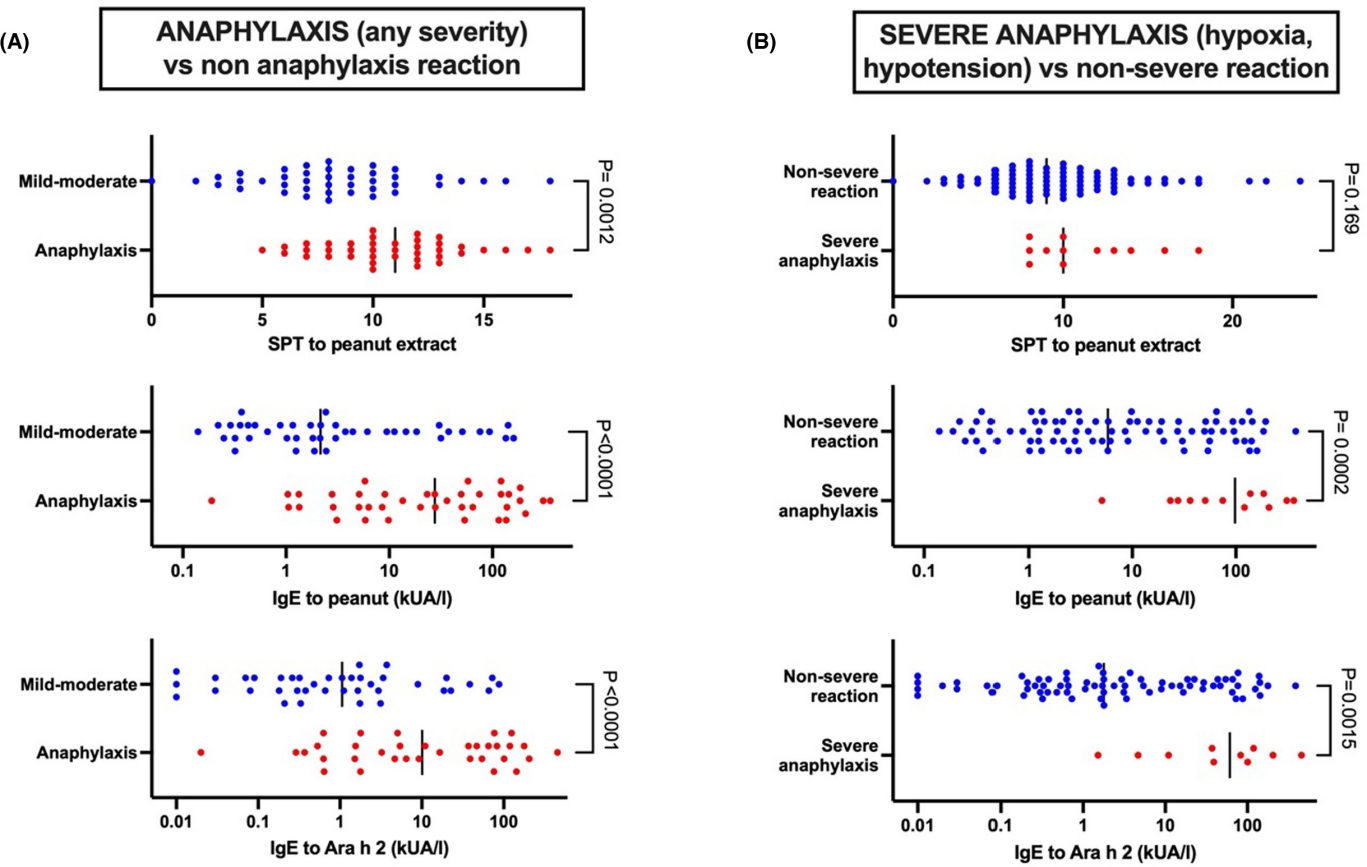
Raw data (skin prick test (SPT), IgE to peanut and Ara h 2) from children with peanut‐induced allergic reactions in the LEAP study cohort.^72^, ^73^, ^74^ There is extensive overlap between (A) those with anaphylaxis and (B) those with severe reactions (Common Terminology Criteria for Adverse Events (CTCAE) Grade 3 reaction) and non‐severe group despite statistical significance between groups

While high‐level IgE sensitization (skin prick test (SPT) wheal and/or specific IgE to food allergens) are usually associated with clinical reactivity, they do not, in general, predict reaction severity or the occurrence of anaphylaxis at food challenge.^3^ Many studies report a correlation between IgE‐sensitization and anaphylaxis (Table 3); however, the overlap in SPT/IgE between those with and without anaphylaxis is so extensive that there is insufficient discrimination to predict risk.^14^, ^15^, ^16^, ^17^, ^18^, ^20^, ^27^, ^28^, ^35^, ^39^, ^40^, ^49^, ^51^, ^57^, ^58^, ^61^, ^62^, ^63^, ^64^, ^65^, ^66^, ^67^, ^68^, ^69^, ^70^, ^71^, ^72^, ^73^, ^74^, ^75^, ^76^ This is clearly demonstrated in well‐curated datasets such as the LEAP study cohort (Figure 2): even IgE to Ara h 2 was not predictive of anaphylaxis at challenge, a finding confirmed elsewhere.^77^ Some authors have overestimated the predictive utility of these tests by including non‐allergic individuals as ‘non‐severe’ reactors in their analyses.^65^, ^69^, ^72^, ^73^, ^74^, ^78^ While this approach is reasonable in the context of predicting *any* clinical reaction in people without a confirmed diagnosis, for severity, clinicians more commonly want to assess risk in patients with *known* allergy. The inclusion of non‐reactors significantly overestimates the specificity and thus, the likelihood ratio of the test (see example in Table 4).

**TABLE 3:** Studies evaluating association of IgE‐sensitization/basophil activation test with reaction severity. Red and orange text indicate a strong or moderate reported positive association, respectively; brown text a weak association and black text no association. Green text indicates an association with lower risk of severe reaction

Study	Allergen	Risk of bias	Predictor of anaphylaxis severity			
			Skin prick test	IgE to whole allergen	Component testing	Basophil activation
Neuman‐Sunshine 2012^27^	Peanut	High		OR 3.29 (1.71–6.32)		
Cianferoni 2012^20^	CM, egg, PN	High	aOR 1.16	aOR 1.01		
van Erp 2013^14^	Peanut	Low	OR 1.05 (0.95–1.15)	OR 1.00 (1.00–1.02)	Ara h 2: OR 1.02 (0.99–1.04)	
Eller 2013^61^	Peanut	Moderate		ρ = .54, *p* < .01	Ara h 2: ρ = .60, *p* < .01	
Klemans 2013^62^	Peanut	Moderate	*r*_s_ = .09, *p* = .43	*r*_s_ = .15, *p* = .15	Ara h 2: *r*_s_ = .23, *p* = .03	
Masthoff 2014^63^, ^64^	Hazelnut	High	Not associated with severity	Not associated with severity	Cor a 1/9 not associated with severity	
Song 2015^35^	Nuts/sesame/seafood	Moderate	*r*_s_ = .24, *p* = .05	*r*_s_ = .33, *p* = .005	Ara h 2: *r*_s_ = .31, *p* = .04	*r*_s_ = .50, *p* < .0001
Kukkonen 2015^65^	Peanut	Moderate		AUC 0.80 (0.71–0.88)	Ara h 2: AUC 0.96 (0.93–0.99)	
Uasuf 2015^66^	Peach	High			Pru *p* 3: OR 69 (2.3–2028) LR 6.3 (4.5–8.8)	
Deschildre 2016^28^	Peanut	Moderate	Weak association with severity	Not associated with severity	Not associated with severity	
Chan 2017^67^	Peanut Egg Sesame	Low	AUC 0.48 (0.34–0.62) AUC 0.57 (0.31–0.83) AUC 0.45 (0.05–0.85)	AUC 0.82 (0.68–0.96) AUC 0.76 (0.59–0.94) AUC 0.70 (0.37–1.0)		
Pettersson 2018^15^	CM, egg, peanut	Low	Predictive for CM and egg (*p* < .02), but not peanut	Predictive for CM and peanut (*p* < .05), but not egg		
Purington 2018^39^	Multiple foods	Moderate		Higher values associated with severity: HR 1.49 (1.19–1.85)		
Chinthrajah 2018^40^	Peanut	Moderate	SPT not predictive (*p* = .96)	IgE not associated with severity (*p* = .13)	IgE to ara h 2 not associated with severity (*p* = .09)	BAT associated with severity (*p* = .004)
Reier‐Nilsen 2018^16^	Peanut	Moderate	No association with severity (data not included in report)			
Yanagida 2018^17^	CM Egg Wheat Peanut	Moderate		CM: aOR 3.19 (1.70–5.98) Egg: aOR 1.99 (1.13–3.53) Wheat: aOR 6.31 (2.37–16.8) Peanut: aOR 4.91 (1.34–17.9)	Bos d 8: aOR 4.03 (1.78–9.12) Gal d 1: aOR 1.61 (1.03–2.53) ω‐5 gliadin: aOR 7.9 (3.1–20.2) Ara h 2: aOR 3.76 (1.12–12.7)	
Datema 2018^57^	Hazelnut	Moderate			*Historical symptoms:* Cor a 1: OR 0.4 (0.2–0.64) Cor a 9: OR 2.9 (1.3–6.3) Cor a 14 OR 4.7 (1.8–12.4) *At food challenge:* Cor a 1: OR 0.14 (0.03–0.55) Cor a 9: OR 10.5 (1.2–91.4) Cor a 14 OR 10.1 (1.1–91.5)	
Palosuo 2018^68^	Egg	Low	Not associated with severity	Not associated with severity	No association for Gal d 1/2/3/4	
Datema 2019^69^	Peanut	Moderate		AUC 0.74 (0.66–0.81)	Ara h 2: AUC 0.80 (0.73–0.87)	
Ballmer‐Weber 2019^70^	Walnut	Moderate		Higher values associated with systemic reactions (*p* <.001); severity not assessed	Jug r 1 (*p* < .001) and Jug r 4 (*p* < .001) associated with systemic reactions; severity not assessed	
Kiewiet 2020^71^	α‐Gal allergy (red meat)	Moderate		Weak association with severity for IgE to beef: AUC 0.61	Weak association with severity for α‐Gal: AUC 0.61	
Santos 2020^72^, ^74^	Peanut	Moderate	For any anaphylaxis: AUC 0.71 (0.59–0.82) For life‐threatening reaction: AUC 0.66 (0.51–0.81)	For any anaphylaxis: AUC 0.75 (0.65–0.86) For life‐threatening reaction: AUC 0.86 (0.76–0.95)	For any anaphylaxis: Ara h 2: AUC 0.76 (0.65–0.87) For life‐threatening reaction: Ara h 2: AUC 0.83 (0.70–0.95)	For any anaphylaxis: AUC 0.69 (0.57–0.81) Life‐threatening reaction AUC 0.88 (0.80–0.96)
Lyons 2021^49^	Walnut	Moderate	OR 1.37 (0.82–2.31)	OR 1.02 (0.99–1.05) AUC 0.74 (0.65–0.83) with or without IgE‐walnut	Jug r 1 OR 1.00 (0.95–1.02) Jug r 4 OR 1.00 (0.93–1.05) AUC 0.81 (0.73–0.89)	
Turner 2021^18^	CM	Low	SPT not predictive	IgE not predictive	Casein: not predictive	
Błażowski 2021^51^	All allergens	Moderate			Gal d 1 (egg) and Ana o 3 (cashew) associated with severity	
Datema 2021^58^	Peanut	Moderate		OR 1.01 (0.99–1.03) AUC 0.72 (0.68–0.75) Prediction not improved by including IgE sensitization	OR 1.08 (0.71–1.63) AUC 0.71 (0.67–0.74) Prediction not improved by including component diagnostics	
Kaur 2021^75^	Peanut	Moderate		Moderate correlation with severity (*r*_s_ = 0.56)	Moderate correlation with severity for Ara h 2 (*r*_s_ = 0.61), not Ara h 1/3 Polysensitization to multiple com‐components associated with severity	
Goldberg 2021^76^	Walnut + Pecan	Moderate	SPT not predictive			Associated with lower respiratory symptoms but not anaphylaxis

**TABLE 4:** Impact of including non‐reactor patients on test sensitivity/specificity/likelihood ratio (LR) when evaluating the diagnostic utility of different biomarkers to predict the occurrence of anaphylaxis or severe reactions to peanut at food challenge, using data from the LEAP study cohort.^72^, ^73^, ^74^ Bold text represent re‐analysis using data from allergic individuals only, in contrast to analyses which included non‐allergic participants as non‐severe reactors. Receiver‐operating characteristic (ROC) curves used to derive area under ROC curve (AUC) are shown in Figure S4

Parameter	Diagnostic cut‐off		AUC	Sensitivity (%) [95% CI]	Specificity (%) [95% CI]	LR
Anaphylaxis (any severity) vs. non anaphylaxis reaction						
BAT (%CD63 basophils)	1.2	Incl. non‐reactors	0.97	95 [83, 99]	87 [84, 89]	7.1
	17	**Reactors only**	**0.69**	**69 [54, 81]**	**57 [42, 71]**	**1.6**
IgE to Ara h 2	0.35 kU/L	Incl. non‐reactors	0.98	94 [81, 99]	95 [94, 97]	21
	1.8 kU/L	**Reactors only**	**0.76**	**71 [54, 83]**	**67 [51, 79]**	**2.1**
IgE to peanut	1 kU/L	Incl. non‐reactors	0.95	97 [87, 100]	83 [81, 86]	5.9
	4.8 kU/L	**Reactors only**	**0.75**	**79 [64, 89** **]**	**67 [52, 79]**	**2.4**
Peanut SPT	8 mm	Incl. non‐reactors	0.98	85 [70, 93]	97 [95, 98]	26.5
	8 mm	**Reactors only**	**0.71**	**85 [70, 93]**	**40 [27, 56]**	**1.4**
Severe reactions [NCI‐CTCAE Grade 3] vs. non‐severe reaction						
BAT [%CD63 basophils]	48	Incl. non‐reactors	0.98	100 [100, 100]	97 [95, 98]	33.3
	48	**Reactors only**	**0.88**	**100 [92, 100]**	**75 [65, 76]**	**4.0**
IgE to Ara h 2	1.4 kU/L	Incl. non‐reactors	0.98	100 [100, 100]	93 [91, 98]	14.3
	4.2 kU/L	**Reactors only**	**0.83**	**90 [60, 90]**	**67 [46, 73]**	**2.7**
IgE to peanut	5 kU/L	Incl. non‐reactors	0.98	100 [100, 100]	90 [87, 98]	10
	22 kU/L	**Reactors only**	**0.86**	**92 [75, 92]**	**74 [51, 75]**	**3.5**
Peanut SPT	8 mm	Incl. non‐reactors	0.96	100 [100, 100]	92 [89, 94]	12.5
	8 mm	**Reactors only**	**0.66**	**100 [42, 100]**	**34 [24, 37]**	**1.5**

Abbreviations: BAT, basophil activation test; SPT, skin prick test.

The best evidence for IgE‐sensitization being suggestive of a higher risk of severe reaction is for Pru p 3 (peach),^66^, ^79^ and the 2S albumins in tree nut allergy,^49^, ^51^, ^57^ although this may be region‐dependent. Some studies report an association between sensitization to specific IgE components and severity; however, often the term ‘severity’ is used to describe *any* systemic reaction (as opposed to local oral symptoms)—even if that systemic reaction does not meet established clinical criteria for anaphylaxis. Thus, some studies which report an association between severity and Ara h 2 for peanut,^80^ Jug r 1/Jug r 4 for walnut,^70^ or Cor a 9/Cor a 14 for hazelnut^63^, ^64^ are actually describing a higher risk of *any* systemic reaction, without differentiating between anaphylaxis and systemic (but non‐anaphylaxis) reactions such as generalized urticaria. This may explain why the diagnostic cut‐offs in some of these studies are similar to those reported to be 95% predictive of *any* clinical reaction.

Birch‐pollen sensitization is associated with less severe reactions in people allergic to peanut^58^ or hazelnut,^57^ probably because this is indicative of Bet v 1‐mediated PFAS, rather than primary sensitization to a food allergen.^81^, ^82^ Importantly, patients with only oral symptoms (often referred to as oral allergy syndrome, OAS) to low levels of allergen exposure must not be assumed to have PFAS on that basis alone: indeed, in the first description of OAS in the literature, almost half of patients with OAS to peanut went on to experience systemic symptoms and anaphylaxis with subsequent exposure.^83^ OAS is therefore not synonymous with PFAS; the latter requires evidence of IgE‐sensitization to cross‐reactive pollens and/or the presence of OAS alone at higher doses of allergen ingestion.^81^, ^82^

While LTP‐mediated allergy can be associated with very severe reactions, LTP‐sensitization without clinical reactivity is common, although there is significant geographical variation.^3^ Data are emerging that polysensitization to pollen panallergens (particularly to Bet v 1 homologues and profilins) in LTP‐sensitized individuals can moderate severity.^59^, ^60^, ^79^, ^84^ Conversely, mono‐sensitization to a single LTP (without other IgE‐sensitization) may be associated with a greater risk of severe reactions in some regions.^60^

#### 2.3.3 Variations in host cellular responses

Some studies report that the basophil activation test (BAT) predicts severity (Table 4),^35^, ^40^, ^70^, ^74^ but as with IgE‐sensitization, reported analyses sometimes overestimate predictive utility by including non‐reactive individuals.^70^ Nonetheless, in a re‐analysis of data from the LEAP study cohort (excluding non‐allergic individuals), BAT was still the best predictor of life‐threatening reactions (such as persistent hypotension and/or hypoxia with decreased level of consciousness) at peanut challenge, although IgE to peanut was not statistically inferior. However, in predicting *any* anaphylaxis (rather than life‐threatening anaphylaxis), BAT was inferior to IgE to peanut, Ara h 2 and even SPT (Table 4 and Figure S4).^70^, ^71^, ^72^

Combining multiple parameters into a predictive model may provide better accuracy. Incorporating component‐resolved diagnostics (but not IgE to whole allergen) into a model improved prediction of more severe symptoms and anaphylaxis for peanut,^48^ hazelnut^49^ and walnut.^41^ Including BAT may further increase predictive utility,^70^ because BAT reflects a more functional readout of the ability of IgE to trigger cellular degranulation—in much the same way that the mast cell activation test has also been shown to correlate with severity.^85^ However, till date, this has only been evaluated in predicting life‐threatening reactions in a cohort where only 12 children had severe reactions.^70^ The extent and frequency of IgE binding (including for specific epitopes) have been reported to correlate with symptom severity at food challenge in some studies,^86^, ^87^, ^88^, ^89^, ^90^ but including IgE avidity and diversity in a prediction model for *any* symptoms in peanut‐allergic individuals did not improve diagnostic utility compared to peanut components.^87^ These data imply that a more complex integration of different allergen‐antibody‐effector cell interactions might confer better severity prediction, but larger and combined datasets reflecting different populations are needed to evaluate whether such models can be helpful in risk‐stratifying patients.

#### 2.3.4 Mastocytosis and elevated baseline mast cell tryptase

There is little evidence that the association of clonal mast cell disorders with severe hymenoptera allergy also applies to food allergy.^91^ Raised mast cell tryptase (MCT) due to hereditary alpha tryptasaemia (HαT) affects around 5% of the population, and is associated with severity in hymenoptera allergy.^92^ However, this has not been demonstrated for food allergy, and raised baseline MCT does not appear to be associated with severity.^31^, ^93^, ^94^, ^95^, ^96^

#### 2.3.5 Immune activation (e.g. intercurrent viral infection)

Data from immunotherapy studies have highlighted that some patients experience a fall in reaction threshold if unwell with viral infections, and this *may* increase the severity of any symptoms experienced.^47^, ^97^, ^98^, ^99^ However, intercurrent illness was not associated with reaction severity in a prospective study in peanut‐allergic adults^45^ or in the European Anaphylaxis Register.^41^ Immune activation for example, due to viral infections, can also cause flares in eczema. Some fatalities due to food‐anaphylaxis have been noted to have active eczema exacerbations,^7^ but data are inconsistent.

### 2.4 Allergen presentation

#### 2.4.1 Key findings

Thirty‐three studies investigated aspects of allergen presentation. Food processing (and the presence of other food ingredients) impact on allergen bioavailability and resulting symptoms, but data are limited. The relationship between dose/level of allergen exposure and reaction severity is unclear. Individuals who react to smaller amounts of allergen are not necessarily at higher risk of anaphylaxis.

#### 2.4.2 Evidence

Anaphylaxis to food is, in general, of lower severity than that due to non‐food triggers,^12^, ^31^, ^37^, ^41^, ^48^ and is associated with predominantly respiratory symptoms.^12^, ^31^ Certain foods may cause more anaphylaxis and life‐threatening reactions than others, even when correcting for prevalence (Table 5). In most regions, peanut/tree nuts are the most common triggers^26^, ^31^, ^33^, ^46^, ^51^, ^100^, ^101^, ^102^, ^103^, ^104^, ^105^ although cow’s and other mammalian milk and seafood are increasingly common causes of fatal and non‐fatal anaphylaxis,^12^, ^32^, ^43^, ^106^, ^107^, ^108^, ^109^, ^110^ responsible for a higher proportion of anaphylaxis than peanut in some regions.^102^ Wheat has been reported to cause more severe reactions (cardiovascular symptoms including loss of consciousness) compared to other foods in adults, possibly due to a greater role of cofactors.^111^ Most case series report that egg is less likely to cause anaphylaxis in children compared with other foods, at least at food challenge,^3^, ^38^, ^112^ although not all studies concur.^10^ Egg is not a common cause of near‐fatal and fatal anaphylaxis.^34^, ^102^, ^103^, ^108^ Foods associated with PFAS often cause anaphylaxis less frequently.^81^, ^102^

**TABLE 5:** Common causes of anaphylaxis by region

	Children	Adults
Europe	**PEANUT** **TREE NUTS** **COW’S MILK** Fish	**PEANUT** **TREE NUTS** Crustacea/fish Cow's milk Wheat Celery root
North America, Australia, New Zealand	**PEANUT** **TREE NUTS** Cow's milk	**PEANUT** **TREE NUTS** **CRUSTACEA**
Asia^a^	Peanut Tree Nuts Cow's milk Egg Wheat	Crustacea/fish Wheat
Africa^a^	Peanut Tree nuts Cow's milk Egg (data from South Africa only)	Peanut Egg (data from Morocco only)
Latin America^a^	Seafood Cow's milk Egg	Seafood Fruit
Near East^a^ (data from Iran, Qatar, Saudi Arabia)	Peanut Tree nuts Cow's milk Egg Fish/seafood	

Note

Foods highlighted in Capitals are the most common causes of fatal reactions reported in those regions.^102^

^a^ No fatality data have been published for Asia.

#### 2.4.3 Dose

**FIGURE 3:**
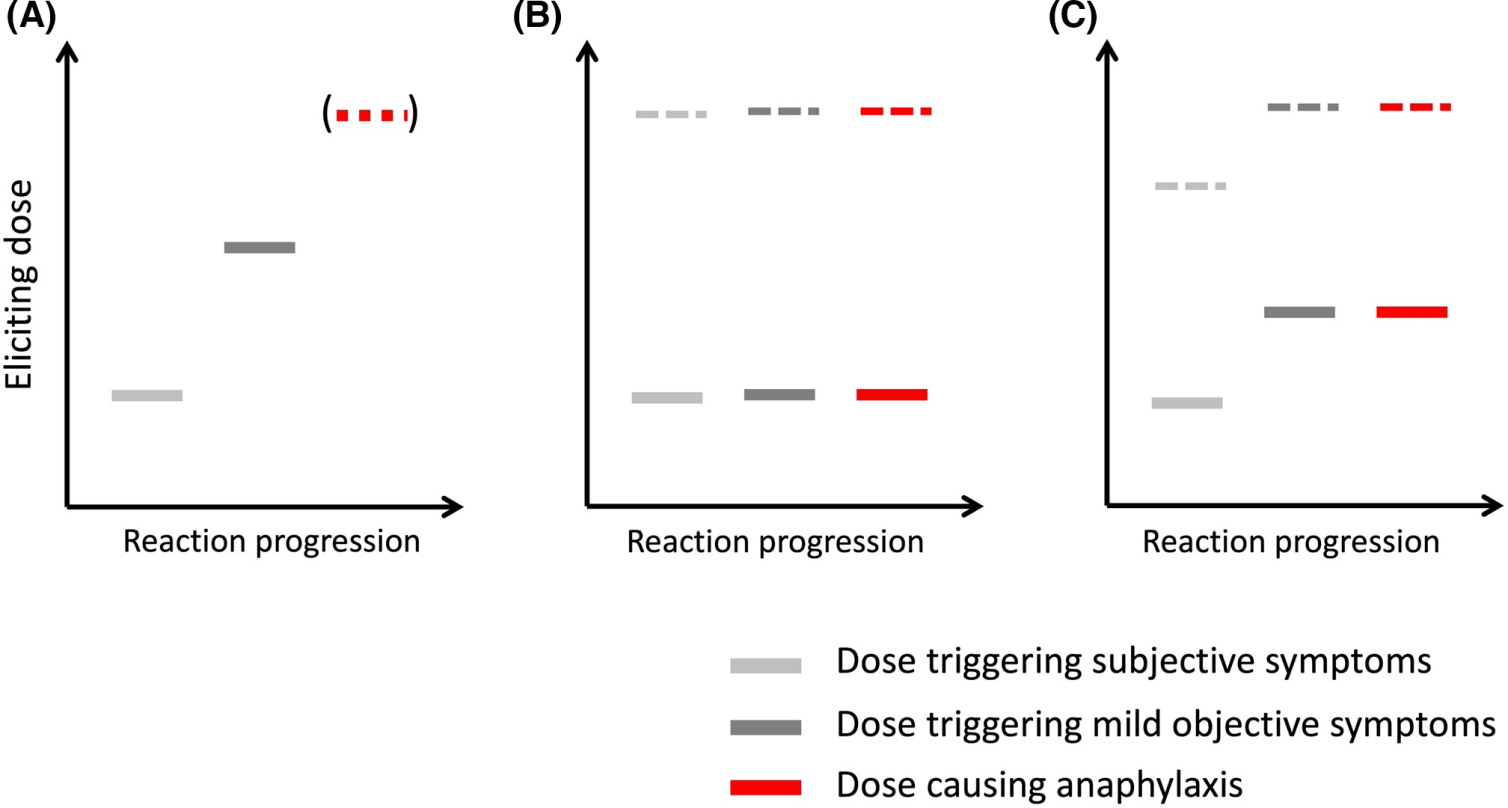
Evolution of symptoms and clinical reactivity at food challenge. Many individuals will experience initially subjective symptoms, with objective symptoms appearing with further doses (A). Anaphylaxis will only develop if the food challenge continues. Others will experience anaphylaxis as their first objective symptom: either at a dose of allergen exposure with no preceding subjective symptoms (B), or with prior subjective symptoms (C), without evidence of a clear dose‐response for symptoms. Note that anaphylaxis can occur at all levels of exposure (both at low levels of allergen exposure, represented by the solid bars, and higher doses indicated by dotted lines). Reproduced under the terms of the Creative Commons Attribution License from reference 9

The relationship between dose/level of exposure and severity is complex and unclear.^5^ For accidental reactions in the community setting, it can be very difficult to accurately determine the degree of allergen exposure associated with an event, particularly those associated with fatal outcomes.^5^ Most individuals experience mild symptoms at food challenge prior to developing more significant (and less mild) symptoms to a dose sufficient to meet challenge stopping criteria^83^—after all, the basic premise of food challenges is the assumption that incremental dosing effectively dose‐limits severity (see Figure 3). In a small study of 27 children reacting to peanut at food challenge, 21 had anaphylaxis but only three as initial presenting symptoms; 13 children presented with initial non‐anaphylaxis symptoms but then developed anaphylaxis with further peanut ingestion.^8^ This pattern has been reproduced elsewhere: that individuals often show a dose‐response between symptoms and level of allergen exposure, at least for the occurrence of *any* anaphylaxis, but this may not be apparent in larger datasets.^9^ In an analysis of 734 double‐blind, placebo‐controlled food challenges from The Netherlands, dose predicted only 4.4% of the variance in reaction severity^15^—in other words, dose was not an important factor. Within a population, there will be a mixture of different allergic phenotypes, with some individuals showing a dose‐response but others not.^9^ Most datasets show that severe reactions can occur at all levels of allergen exposure (which further obscures the dose‐severity relationship in larger datasets),^29^ although significant symptoms are very uncommon to sub‐milligram levels of protein.^113^, ^114^ Finally, in the challenge setting, potential severity may be dose‐limited; so, these data may not be applicable to accidental reactions in the community.^9^ This is important, as controlling exposure is a key modifiable factor for food businesses aiming to provide safe food for people with food allergy.

For any given individual patient, therefore, the absence of prior anaphylaxis may be due to insufficient allergen exposure causing previous reactions, (rather than the patient being at an inherently lower risk).^3^, ^5^, ^6^, ^8^, ^9^, ^12^ Therefore, a history (or not) of anaphylaxis is a poor predictor of future anaphylaxis.^6^, ^10^, ^11^, ^12^, ^13^, ^14^, ^15^, ^16^, ^17^, ^18^, ^19^, ^20^ Reassuringly, there is no robust evidence that food‐allergic individuals who react to very low levels of allergen are at greater risk of anaphylaxis.^3^, ^115^, ^116^, ^117^, ^118^

A history of reaction to only relatively large exposures (with no or minimal symptoms to smaller doses) may be useful in informing the approach to allergen avoidance in any given individual, particularly given more recent data suggesting that reaction thresholds for an food‐allergic person are fairly reproducible under typical challenge conditions, at least for peanut and cow’s milk.^116^, ^118^

#### 2.4.4 Relevance of the food matrix

The impact of the food matrix is well‐established for egg and cow's milk: up to two‐thirds of children allergic to egg and cow's milk can tolerate the allergen when in a baked matrix (such as cake), probably because of heat‐induced 3D structural changes in the protein affecting the ability of IgE to bind to the allergenic epitopes.^3^ Those allergic to ‘baked egg’ or ‘baked milk’ often report delayed and more severe reactions.^119^ perhaps because the matrix slows gastrointestinal absorption, allowing more allergen to be eaten prior to onset of symptoms and thus increasing severity. At least one fatal reaction to baked milk has been reported at in‐hospital food challenge.^120^ It has been suggested that the lack of tolerance to baked milk may be a marker of more severe allergy to cow’s milk,^121^ although this has not been evaluated prospectively. The fat content of the food matrix can impact on the dose threshold triggering symptoms, and the severity of those symptoms, at least for peanut^122^, ^123^ and hazelnut,^123^ although not for egg.^124^ Food processing has a significant impact on the bioavailability of egg and peanut allergens *in vitro*,^125^, ^126^, ^127^ but the data are currently lacking to evaluate the clinical relevance of these findings.

### 2.5 Risk‐taking and other behaviours

#### 2.5.1 Key findings

Risk‐taking is a clear concern in managing people with food allergy, but there is insufficient evidence that risk‐taking is a major factor in fatal outcomes. This does not negate the need to address concerns over risk‐taking in specific individuals. Cofactors such as exercise can lower reaction thresholds in 10–20% of individuals, but with the notable exception of food‐dependent exercise‐induced anaphylaxis (FDEIA), are unlikely to worsen outcomes in most food‐allergic individuals.

#### 2.5.2 Impact of age

Epidemiological data suggests an age‐related increase in severe food‐anaphylaxis in adolescents and young adults.^1^, ^32^, ^33^, ^34^, ^35^, ^36^, ^37^, ^43^, ^106^, ^107^, ^108^ Often, this is assumed to be due to risk‐taking behaviours such as deliberately eating risky food or refusing to carry rescue medication.^128^, ^129^ However, an analysis of national fatal anaphylaxis data from the UK reported that this age‐related increase in near‐fatal and fatal food‐anaphylaxis persists well into the fourth decade of life.^2^, ^34^ The authors suggested that there may be an ‘age‐specific vulnerability to severe outcomes from food‐induced allergic reactions in the second and third decades’.^34^ Adolescents use a variety of different strategies to manage risk, and most teenagers manage their food allergies well.^129^, ^130^ Risk‐taking can be a deliberate act by adolescents to increase their independence,^3^ but can be mitigated by ‘transitioning’ children to self‐care (from around age 11 years). Specific educational interventions targeting teenagers and young adults are unlikely to be harmful, but there is an absence of evidence as to whether such strategies reduce the risk of severe outcomes.^130^

#### 2.5.3 Exercise

Exercise is the most well‐described cofactor in food‐anaphylaxis, reported in 10–20% of cases.^47^, ^131^, ^132^ It is also a common cofactor in adverse events due to oral immunotherapy.^47^, ^97^, ^98^, ^99^, ^133^ Conventional thinking has distinguished between ‘typical’ food allergy where exercise may exacerbate symptoms, and food‐dependent EAI, where allergic symptoms only occur in the context of exercise, typically 2–4 h after consumption of the relevant food. The most commonly described trigger is wheat, associated with IgE‐sensitization to omega‐5‐gliadin (wheat‐dependent EIA, WDEAI).^134^, ^135^ However, a recent study has challenged this distinction. Christensen et al. reported 71 adults with a history of WDEIA who underwent controlled challenges: 26 reacted to very high doses of wheat in the absence of exercise, while 21 *only* reacted in the presence of exercise. The authors, thus implying that the primary impact of exercise in WDEIA is to reduce the reaction threshold resulting in affected individuals developing allergic symptoms to more typical levels of wheat exposure in the context of exercise.^135^ The authors suggest that many individuals with WDEIA are wheat‐allergic even at rest, but tolerate normal levels of wheat ingestion in the absence of exercise due to a very high reaction threshold. However, in the presence of exercise, there is a significant drop in reaction threshold resulting in symptoms to more typical levels of wheat exposure, resulting in reactions. The authors also observed a greater tendency towards *any* anaphylaxis with exercise, which might imply a relationship between dose and resulting reaction severity.^135^

A randomized controlled study in 73 peanut‐allergic adults reported that exercise can reduce an individual's reaction threshold.^13^ However, this was based on dose‐distribution modelling which may have over‐estimated the effect. Analysis of the raw data shows that the average change was more modest.^136^ Overall, 47% of participants had no change in threshold, while in 36% this was limited to a single dosing increment (e.g. 100–30 mg)—well within the inherent variability in reaction thresholds reported for peanut allergy.^116^ Only 12% had a more significant fall in threshold (>0.3 log, equivalent to 1 dosing increment at food challenge). Thus, the impact of exercise does not seem to be important in the majority of peanut‐allergic individuals. Unfortunately, no data relating to symptom severity in this cohort have yet been published.^13^

#### 2.5.4 Alcohol consumption

Alcohol consumption: evidence is mixed. A large registry study reported it to be a possible cofactor in a minority (3%) of anaphylaxis events^131^ whereas a prospective series of accidental reactions in food‐allergic adults reported alcohol consumption in 16% of reactions.^45^ In a sub‐cohort of adults with WDEIA reported by Christensen et al.,^135^ alcohol did not result in a significant change in either reaction threshold or severity compared with baseline challenge at rest.^137^ While intoxication can cause risk‐taking and impair self‐management, alcohol can also activate effector cells (mast cells, basophils) *in vitro*, potentially exacerbating severity via a biological mechanism.^3^

#### 2.5.5 Environment

A disproportionately higher number of fatal anaphylaxis events occur when food‐allergic individuals are away from home for example, on vacation.^7^ This risk may be mitigated through focused patient information, such as translation cards.

### 2.6 Concomitant medications

#### 2.6.1 Key finding

Based on 6 studies, it is likely that β‐blockers and/or angiotensin‐converting enzyme (ACE) inhibitors can increase reaction severity, although any impact seems to be less than other factors such as age, exposure to a non‐food trigger or mast cell disease.

#### 2.6.2 Evidence

The impact of medication on severity is difficult to disentangle, because the underlying reason for prescription (e.g. cardiovascular disease, age) is likely to be a confounder.^3^, ^37^ Few studies have attempted to adjust for this. In a large retrospective study of emergency presentations for anaphylaxis, ACE inhibitor prescription was associated with increased odds of severe reaction after adjusting for cardiovascular disease and age. There was no increased risk for antidepressants, β‐blockers, alpha‐adrenergic blockers or angiotensin II receptor antagonists.^33^ An association between β‐blockers or ACE inhibitors and severity has also been reported in prospective^48^ and retrospective^41^, ^131^ case series. Acetylsalicylic acid (aspirin) and other non‐steroidal, anti‐inflammatory drugs (NSAIDs) have also been reported to increase severity in some analyses,^48^, ^137^ but not in others.^10^, ^41^, ^134^

A meta‐analysis of 15 observational studies reported that β‐blockers (OR 2.2, 95% CI 1.3–3.8) or ACE inhibitors (OR 1.6, 95% CI 1.1–2.2) increased (all‐cause) anaphylaxis severity; however, the authors were unable to adjust for underlying cardiovascular disease or any differences between food and non‐food triggers.^138^ Age is strongly associated with cardiovascular disease and use of β‐blockers, ACE inhibitors, angiotensin receptor blockers or other vasodilators in patients presenting with anaphylaxis^31^; so, it is unclear whether these medicines have a direct impact on severity or if the association is due to cofounding (e.g. by underlying cardiovascular disease). In a prospective series of accidental allergic reactions to food, prescription of β‐blockers, ACE inhibitors, angiotensin receptor blockers were not more common in those experiencing more severe reactions.^45^

Cofactors such as NSAIDs and exercise may influence severity through an impact on allergen absorption.^3^ The gastrointestinal epithelial barrier can be impaired in food allergy, although consistent evidence is lacking.^139^ Intestinal permeability is not predictive of food allergy,^140^ but may have a key role in WDEIA.^141^ Measuring food proteins in serum following ingestion is difficult.^139^ Nonetheless, greater absorption kinetics for peanut has been reported peanut‐allergic subjects compared with non‐allergic controls: significantly lower amounts of peanut (30mg protein) were required to detect Ara h 6 in serum samples in peanut‐allergic individuals, and for any given peanut dose ingested, higher Ara h 6 was found in sera from peanut‐allergic participants versus controls.^139^ Whether this phenomena reflects antibody‐mediated facilitated absorption is unclear. Allergen absorption kinetics may be a key determinant of severity in a murine model of peanut anaphylaxis^142^; thus, further in‐human studies are warranted.

### 2.7 Non‐modifiable host factors

Many studies report an association between age and risk of food‐anaphylaxis of *any* severity,^20^, ^28^, ^33^, ^37^, ^44^, ^104^, ^110^, ^114^ but not all.^14^, ^27^, ^100^ Anaphylaxis is most commonly reported in preschool children age 0–4 years, although severe or fatal outcomes in this age group are rare.^34^, ^108^ The age group at greatest (although still very low) risk of near‐fatal and fatal anaphylaxis to food is in adolescents and adults up to age 40 years.^2^, ^34^, ^108^ Males may be at slightly higher risk of severe anaphylaxis, both pre‐ and post‐puberty.^143^ At least one fatal series has reported male sex to be a risk factor.^106^ Till date, no clear genetic associations have been identified conferring a greater risk of severe allergic reactions to food.^3^ Specific HLA haplotypes have been reported for WDEIA,^144^ but for other food allergy phenotypes, data are very inconsistent.

### 2.8 Management of allergic reaction

**FIGURE 4:**
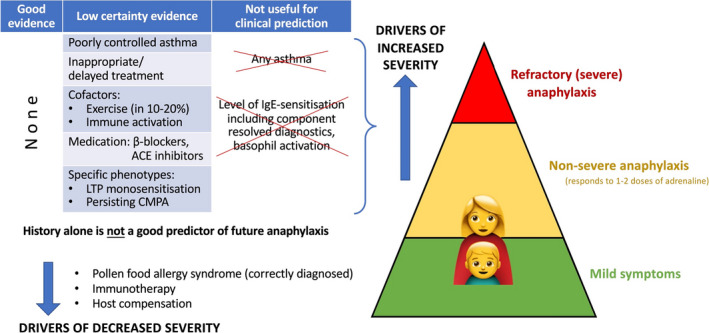
Factors which influence the severity of anaphylaxis. Elicitors and cofactors may act synergistically making anaphylaxis more likely. The natural ability of the body to compensate for anaphylaxis in combination with therapeutic measures will moderate the severity of reaction. CMPA, cow's milk protein allergy; LTP, lipid transfer protein

Delays in symptom recognition and treatment of anaphylaxis have been associated with more severe outcomes in anaphylaxis, including need for intensive care and length of hospital stay.^3^, ^42^, ^48^, ^100^, ^145^, ^146^, ^147^ Whether delays in adrenaline treatment also increase the risk of biphasic anaphylaxis is less clear.^145^, ^146^, ^147^, ^148^ There is no evidence that treating non‐anaphylaxis reactions with adrenaline helps prevent progression to anaphylaxis.^147^, ^149^ Observational data from food challenges in peanut‐allergic adults have shed new light on homeostatic mechanisms which can compensate for anaphylaxis and prevent severe outcomes.^150^, ^151^ One hypothesis is that individuals at greater risk of severe reactions may be less able to compensate for an allergic insult (e.g. through endogenous catecholamine production) (Figure 4),^42^, ^150^ but this needs further evaluation.

## 3 SEVERITY CONSIDERATIONS: FOOD PROTEIN INDUCED ENTEROCOLITIS SYNDROME

We identified only 2 papers eligible for inclusion about FPIES. One noted that poor weight gain was more common where the trigger was cow's milk or banana. Why banana might be a risk factor is unclear.^152^ Another study of 222 FPIES food challenges had only four severe reactions, which precludes any meaningful analysis.^153^

## 4 LIMITATIONS OF THIS REVIEW

We chose to focus on IgE‐mediated food allergy and FPIES, as these are arguably the only food allergy phenotypes with clear diagnostic criteria associated with acute, life‐threatening reactions. However, since only two papers relating to FPIES were identified for inclusion, the majority of this review relates to IgE‐mediated food allergy and anaphylaxis. The majority of included studies were from Europe and North America, thus, our conclusions require confirmation in food‐allergic people from other regions. The extensive heterogeneity in severity definitions applied to food allergy, and their highly variable usage by individual studies, precluded a more quantitative analysis of the evidence.

## 5 CONCLUSIONS

It is vital that food‐allergic individuals receive reliable and accurate information to help them self‐manage their condition. Our comprehensive review suggests that there is much left to learn about risk factors for anaphylaxis and life‐threatening reactions (Table 1). Absence of prior anaphylaxis does not exclude future risk of anaphylaxis, and history alone is not a good predictor because severity depends on multiple factors including dose of exposure and the presence or absence of cofactors (e.g. exercise, concurrent viral infections and some medications).

Importantly, our review challenges widely‐held (but evidence‐poor) conventions that asthma or degree of IgE‐sensitization are useful predictors. Our meta‐analysis shows that a diagnosis of asthma in itself is unlikely to be a significant risk factor if asthma control is satisfactory.

Higher levels of IgE‐sensitization are associated with a history of anaphylaxis, but in practice, biomarkers of IgE‐sensitization are not helpful in predicting severity. Clinicians may incorrectly interpret low levels of IgE‐sensitization as implying a lower risk of anaphylaxis and provide incorrect information to their patients as a result, while those with high IgE‐sensitization are wrongly counselled that they are at high risk of severe reactions. Similarly, most individuals with food allergy experience oral symptoms to low doses: thus, the occurrence of oral symptoms *alone* to low allergen exposure must not be assumed to imply PFAS, and therefore, a lower risk of anaphylaxis.

Individuals with food allergy must understand that they can experience anaphylaxis in the future, even if they appear to be at low risk. All patients with food allergy need to be able to recognize and appropriately self‐manage anaphylaxis. It is necessary to weigh up the whole clinical scenario carefully when evaluating risk. A history of previous anaphylaxis would place an individual in a risk group where access to self‐injectable adrenaline is indicated. Healthcare professionals might consider that other factors, such as being an adolescent or young adult, also move an individual into a higher risk category. Those with food allergy need to be informed that future reactions may be of differing severities. They need to be counselled as to the potential for some cofactors to increase reaction severity, and if they have coexisting asthma, it is prudent to optimize asthma control.

Our inability to accurately risk‐stratify food‐allergic individuals is an important knowledge gap. As a consequence, many patients are prescribed self‐injectable adrenaline yet most do not use it, even when indicated. Being able to predict those most at risk would help to target interventions towards those at greatest risk. Large longitudinal population‐based studies are needed to identify specific factors which predict future risk. This will help achieve a better understanding of the underlying mechanisms of severe allergic reactions, from initiation to end‐organ involvement.

## CONFLICT OF INTEREST

Dr Turner reports personal fees from Aimmune Therapeutics, DBV Technologies, Allergenis, UK Food Standards Agency and ILSI Europe; grants from National Institute for Health Research (NIHR)/Imperial Biomedical Research Centre, UK Medical Research Council, UK Food Standards Agency, End Allergies Together, Jon Moulton Charity Trust, outside the submitted work. Prof Halken reports speaker/chair fees from Nestlé Purina and ABIGO Pharma A/S. Dr Muraro reports speaker fees from Aimmune Therapeutics, DBV, and Nestlé Purina; and is an advisory board member/fees from Regeneron IDMC. Dr Deschildre reports personal fees from ALK, Stallergènes‐Greer, GSK, Novartis, Sanofi, Regeneron, Bohringer Ingelheim, Aimmune therapeutics, DBV technologies, Nestlé Health Science, Nutricia, outside the submitted work. Dr Ballmer‐Weber reports personal fees from ALK, Allergopharma, ThermoFisher, Menarini, Sanofi, Novartis. Prof. van Ree reports personal fees from HAL Allergy, ALK, Citeq, Angany, ThermoFisher, Reacta Healthcare, and Mission MightyMe; grants from European Commission, Dutch Science Foundation, and Health Holland, outside the submitted work. Prof. Worm declares the receipt of honoraria or consultation fees by the following companies: ALK‐Abelló Arzneimittel GmbH, Mylan Germany GmbH, Leo Pharma GmbH, Sanofi‐Aventis Deutschland GmbH, Regeneron Pharmaceuticals, DBV Technologies S.A, Stallergenes GmbH, HAL Allergie GmbH, Allergopharma GmbH & Co.KG, Bencard Allergie GmbH, Aimmune Therapeutics UK Limited, Actelion Pharmaceuticals Deutschland GmbH, Novartis AG, Biotest AG., AbbVie Deutschland GmbH & Co. KG and Lilly Deutschland GmbH, outside the submitted work. Prof Zuberbier reports personal fees from Bayer, FAES, Novartis, Henkel, AstraZeneca, AbbVie, ALK, Almirall, Astellas, Bencard, Berlin Chemie, HAL, Leti, Meda, Menarini, Merck, MSD, Pfizer, Sanofi, Stallergenes, Takeda, Teva, UCB, Henkel, Kryolan, L’Oréal, outside the submitted work. Prof Roberts reports grants from UK Food Standards Agency, DBV and the European Union. The other authors have no relevant conflicts of interest to declare.

## AUTHOR CONTRIBUTIONS

All authors conceptualized the review. PJT, SA, BBW, ABC, AD, JG, AM and RvR extracted the data. PJT and NP undertook meta‐analysis. All authors contributed to the analysis and data interpretation. PJT drafted the manuscript, which was revised, reviewed and approved by all authors. PJT and GR oversaw the process and acted as arbitrators.

## Supporting information

Supplementary Material
