# Supplementary material for: Risk factors for severe reactions in food allergy: Rapid evidence review with meta‐analysis
Source: Allergy. 2022 Apr 28;77(9):2634–52. doi: 10.1111/all.15318 (PMC9544052; doi:10.1111/all.15318)

ONLINE SUPPLEMENT

Risk factors for severe reactions in food allergy: rapid evidence review with meta-analysis

Paul J. Turner^1^*, Stefania Arasi^2^, Barbara Ballmer-Weber^3^, Alessia Baseggio Conrado^1^, Antoine Deschildre^4^, Jennifer Gerdts^5^, Susanne Halken^6^, Antonella Muraro^7^, Nandinee Patel^1^, Ronald Van Ree^8^, Debra de Silva^9^, Margitta Worm^10^, Torsten Zuberbier^10^, Graham Roberts^11,12^; on behalf of the Global Allergy and Asthma European Network (GA2LEN) Food Allergy Guideline Group

**Affiliations:**

^1^National Heart & Lung Institute, Imperial College London, London, United Kingdom;

^2^Translational Research in Paediatric Specialities Area, Division of Allergy, Bambino Gesù Children's Hospital, IRCCS, Rome, Italy;

^3^Clinic for Dermatology and Allergology, Kantonsspital St. Gallen, St. Gallen, Switzerland and Department of Dermatology, University Hospital Zürich, Zürich, Switzerland;

^4^CHU Lille, Univ. Lille, Pediatric Pulmonology and Allergy Department. Hôpital Jeanne de Flandre, 59000 Lille, France;

^5^Food Allergy Canada, Toronto, ON, Canada;

^6^Hans Christian Andersen Children’s Hospital, Odense University Hospital, Odense, Denmark;

^7^Food Allergy Centre, Padua General University Hospital, Padua, Italy;

^8^Amsterdam University Medical Centers, location AMC, Departments of Experimental Immunology and of Otorhinolaryngology, Amsterdam, The Netherlands;

^9^The Evidence Centre, London, United Kingdom;

^10^Division of Allergy and Immunology, Dpt Dermatology, Venerology and Allergy, Charité, Universitätsmedizin Berlin, Germany;

^11^NIHR Southampton Biomedical Research Centre, University Hospital Southampton NHS Foundation Trust; Faculty of Medicine, University of Southampton, Southampton, UK;

^12^The David Hide Asthma and Allergy Research Centre, St Mary's Hospital, Isle of Wight, UK.

**SUPPLEMENTARY METHODS**

**Search strategy**

The methods and analyses were planned *a priori*. We searched MEDLINE, EMBASE and the Cochrane Register of Controlled Trials, including all primary records from 1 January 2010 until 31 August 2021. We used the following search strategy:

1. sever*.mp. [mp=ti, ab, hw, tn, ot, dm, mf, dv, kw, fx, dq, nm, kf, ox, px, rx, ui, sy] AND (food or peanut or milk or egg or wheat or LTP or nut or fish or seafood or crustac*).mp. [mp=ti, ab, hw, tn, ot, dm, mf, dv, kw, fx, dq, nm, kf, ox, px, rx, ui, sy] AND allergy.mp. [mp=ti, ab, hw, tn, ot, dm, mf, dv, kw, fx, dq, nm, kf, ox, px, rx, ui, sy]
2. limit 1 to human
3. (systematic or review or randomised or randomized or control* or placebo or cohort or observational or registry).mp. [mp=ti, ab, hw, tn, ot, dm, mf, dv, kw, fx, dq, nm, kf, ox, px, rx, ui, sy]
4. 2 and 3

We also reviewed reference lists of included studies and review articles to identify other relevant studies. There were no language restrictions.

**Study selection**

Table S1 lists eligible study types. The inclusion criteria were:

Population: People with IgE-mediated food allergy or FPIES, confirmed either by food challenge or clinician-assessed history of severe reaction (e.g. anaphylaxis).

Study focus: Any predictor associated with more severe outcomes due to acute reactions: hospitalisation, intensive care admission, death.

Outcomes: severe anaphylaxis; morbidity or mortality

Abstracts were independently screened by at least two authors (PJT, SA, ABC) to identify potentially relevant studies. The full text of shortlisted studies were screened by at least 2 authors (clinicians, allied health professionals and patient representatives) to reach consensus about which papers to include. We included only published, peer-reviewed full papers or research letters, and excluded conference abstracts. Where repeated reports of the same study were identified, we included the most up-to-date or detailed report.

**Data extraction**

Data were extracted from eligible papers by pairs of authors and then checked by another author (PJT or SA). Authors extracted study characteristics and outcomes independently using a bespoke form.

**Risk of bias of individual studies**

Pairs of authors independently assessed the risk of bias in individual studies. Randomised controlled trials were assessed using the Cochrane Risk of Bias tool 2 (ROB2). This focuses on bias due to the (i) randomisation process; (ii) assignment / deviations from intended group; (iii) missing data; (iv) outcome measurement; and (v) reporting. Each was rated as at low, moderate or high risk of bias.

Other studies were evaluated using the approach of Hoy et al,^S2^ which assesses internal and external validity. Internal validity reflects the degree of systematic data collection and potential bias due to how this data was obtained (e.g. direct from patients, contemporaneous medical notes, historical case notes). External validity assesses for whether selection bias impacts on whether the study data are generalizable to the overall food-allergic population. Arbitration was available if needed from a senior clinician but there was agreement in the risk of bias assessments.

**Synthesis of results**

We synthesised most findings narratively because the data were insufficient or too heterogeneous to undertake meta-analysis. All co-authors developed conclusions by consensus, recognising any potential conflicts of interest, which were declared in advance.

For the data on asthma, we undertook random effects meta-analysis. In brief, severity outcomes were extracted as a binary outcome and odds ratios (OR) calculated, where these were not reported by individual studies. Study heterogeneity was assessed using the I2 statistic. Meta-analysis was performed using Meta Package, R project, version 4.0.3a (random-effects model, REML). We included a sensitivity analysis limited to studies reported unadjusted OR (Figure S1). Funnels plots were used to visually assess for small-study effects were performed by using funnel plots to assess asymmetry and Egger tests (Figure S2, S3).

**SEARCH RESULTS**

3175 studies were screened and 88 studies were included (Table S2). Details of included studies are in Table S3, with risk of bias assessment reported in Table S4. Studies excluded at full text screening stage are reported in Table S5, together with the reason for exclusion.

**SUPPLEMENTARY REFERENCES**

1. Collins AM, Coughlin D, Miller J, Kirk S. The Production of Quick Scoping Reviews and Rapid Evidence Assessments: A How to Guide. Dec 2015. Available at assets.publishing.service.gov.uk/government/uploads/system/uploads/attachment_data/file/560521/Production_of_quick_scoping_reviews_and_rapid_evidence_assessments.pdf (accessed 25 June 2021)
2. Hoy D, Brooks P, Woolf A, et al. Assessing risk of bias in prevalence studies: modification of an existing tool and evidence of interrater agreement. J Clin Epidemiol. 2012;65(9):934-939.

**Table S1**: Inclusion criteria for studies (all in humans)

| **Study type:** | **Restrictions:** |
| --- | --- |
| Systematic reviews | None |
| Randomised controlled trials or controlled clinical trials | None |
| Prospective cohort studies with single or double-blinded placebo-controlled food challenges | Minimum 50 participants with positive challenges |
| Other case series/cohort studies | Minimum 100 participants with   - open food challenges to confirm diagnosis, or clinician-assessed history of anaphylaxis - clinician-assessed history of FPIES reactions   Otherwise minimum 500 participants e.g. patient registries |
| Case series with n≥15 | Limited to reports of fatal or near-fatal anaphylaxis, or FPIES reactions requiring intensive care |

**Table S2: Number of studies screened, included and excluded**

| Number of potential studies identified by database searches | 3142 |
| --- | --- |
| Number of additional potential studies identified through other sources | 33 |
| Total number of studies screened once duplicates were removed | 3175 |
| Number of studies shortlisted for full text review | 105 |
| Number of studies excluded after full text review | 17 |
| Number and type of studies included | Total 88:   - 4 Systematic Reviews - 9 RCT* - 75 Other |

*includes prospective cohort studies with double-blind, placebo-controlled food challenges where severity was a prespecified outcome measure.

**Table S3**: Included studies

| Citation | Study type | Risk of bias | Country | Funding source | Total no. participants | | Age | Allergy type | Severity Definition | Risk factor(s) assessed |
| --- | --- | --- | --- | --- | --- | --- | --- | --- | --- | --- |
|  |  |  |  |  | Overall | “severe” group |  |  |  |  |
| Taylor 2010^115^ | DBPCFC  Retrospective | Moderate | France | Industry and non-industry | 286 | 40 | ≤48 y  Median 7.0 y | Peanut | Astier Grade 4/5 | Eliciting dose at challenge by history of prior anaphylaxis |
| Pastorello 2011^84^ | Prospective cohort | High | Italy | Industry and non-industry | 148 | 72 | 13-62 y  Median 37 y | Peach | Systemic symptoms | Severity of historical reactions |
| Calvani 2011^10^ | Retrospective case series (consecutive recruitment) | Moderate | Italy | Industry | 163 included  (21 excluded: incomplete data) | 36 | ≤18 y  Median 4y | Any food | Sampson grade 4/5 | Age, sex, comorbidities, trigger, cofactors |
| Huang 2012^100^ | Retrospective  case series | High | USA | Non-industry | 192  (152 to food) | 15 | ≤18 y  Median 8 y | Any food | Brown Grade 3 | Hospital admission +/- ICU |
| Neuman-Sunshine 2012^27^ | Retrospective  case series | High | USA | Non-industry | 782 | 164 | ≤16 y | Peanut | CVS/resp or symptoms from 3+ organs | IgE sensitisation, age, asthma |
| Dang 2012^77^ | Prospective DBPCFC (RCT) | Low | Australia | Non-industry | 100 | 4 | Age 1 y | Peanut | Anaphylaxis (ASCIA) | IgE sensitisation |
| Eller 2012^114^ | Open and blinded FC, retrospective | Moderate | Denmark | Internal | 487 | Not stated | 6m – 74 y  [0.5–73.5] | Egg, milk, hazelnut, peanut | Sampson grade 4/5 | Age, threshold |
| Nguyen-Luu 2012^6^ | Retrospective case series | High | Canada | Non-industry | 1411 | N/A | Children, mean 7.1 y | Peanut | Severe as per Hourihane 1997 | Severity of index vs subsequent reaction |
| Rolinck-Werninghaus 2012^29^ | Prospective DBPCFC (RCT) | Moderate | Germany | Internal funds | 869 | 51 | ≤16 y  Median 1 y | Egg, milk, soy, wheat | Sampson grade 4/5 | IgE, age, history of wheezing, dose |
| Cianferoni 2012^20^ | Open FC, retrospective | High | USA | Not stated | 983 | 111 | Mean 5 y | Egg, milk, peanut | 2+ organs require -ing treatment | IgE sensitisation, age, prior reaction |
| Vetander 2012^53^ | Retrospective case series | Moderate | Sweden | Internal | 371 | 128 | ≤17 y  Mean 6 y | All foods | EAACI 2007 | Age, sex, trigger, comorbidities |
| Eller 2013^61^ | Open and blinded FC, retrospective | Moderate | Denmark | Industry + internal | 175 | Not stated | 1-26 y  Mean 5.6 y | Peanut | Sampson grade 4/5 | Age, eliciting dose, IgE |
| Masthoff 2013^63^ | DBPCFC, retrospective | High | Netherlands | Industry | 161 | 79 | Median  7y (children)  27y (adults) | Hazelnut | Any objective symptoms | IgE sensitisation |
| van Erp 2013^14^ | DBPCFC, retrospective | Low | Netherlands | Internal | 109 | 24 | Median 6.7y (IQR 5-9.5) | Peanut | Sampson grade 4/5 | age, sex, asthma, sIgE, prior reaction |
| Brown 2013^31^ | Prospective cohort | Low | Australia | Non-industry | 412  131 food | 97  19 food | 3-99 years Median 36 y  IQR 24-50y | All foods | Brown Grade 3 | Age, trigger comorbidities,  mast cell tryptase |
| Libbers 2013^124^ | DBPCFC, retrospective | Moderate | Netherlands | Not stated | 59 | Not stated | Children | Egg | Study-defined | Food matrix on rection severity |
| Klemens 2013^62^ | DBPCFC, retrospective | Moderate | Netherlands | Internal | 93 | Not stated | Mean 30 y (sd ± 12.5) | Peanut | Adapted from Mueller grade 3/4 | IgE sensitisation |
| Mulla 2013^30^ | State-wide hospital data | Moderate | USA | Internal | 2410  (all trigger) | Not stated | Median 50 y | All foods | ICU or mech-anical ventilation | Asthma |
| Johnson 2014^101^ | Retrospective case series | Moderate | Sweden | Non-industry + internal | 578 | 239 | Median 5.9 y IQR 2.3-12y | All foods | NIAID with adrenaline treatment | Food trigger |
| Vetander 2014^11^ | Retrospective case series | Moderate | Sweden | Internal | 358 | 20 | ≤17 y  Mean 5 y | All foods | EAACI 2007 | Severity of patients having 2+ episodes of anaphylaxis |
| Clark 2014^33^ | Retrospective case series | High | USA | Not stated | 11,972  (20% food) | 2622 | Adults + children | All foods | Hospital/ICU admission | Age, comorbidities |
| Jerschow 2014^106^ | Fatality case series | Low | USA | Internal | 164 | 164 | Adults + children | All foods | Fatal anaphylaxis | Age, ethnicity, sex |
| Xu 2014^32^ | Fatality case series | Moderate | Canada | Internal | 40 | 40 | 9-78 y  Mean 32 y | All foods | Fatal anaphylaxis | Age, asthma, trigger, carriage of epinephrine |
| Nassiri 2015^131^ | Anaphylaxis registry | High | Europe | Not stated | 1222 | 116 | Adults + children | All foods | Mueller | ACE inhibitors,  beta-blockers |
| Turner 2015^34^ | Fatality case series | Moderate | UK | Non-industry | 124 | 124 | Adults + children | All foods | Fatal anaphylaxis | Age, sex, trigger, asthma |
| Song 2015^35^ | Prospective DBPCFC (RCT) | Moderate | USA | Industry and non- industry | 58 | Not stated | 12-45 y | Nuts, seafood, sesame | Sampson Grade | IgE sensitisation, BAT |
| Kukkonen 2015^65^ | Prospective DBPCFC (RCT) | Moderate | Finland | Non-industry | 69 | 25 | 6-18y | Peanut | Hourihane 2005 | IgE sensitisation |
| Francuzik 2015^42^ | Anaphylaxis registry | Moderate | Europe | Internal | 5765 | 116 | Adults + children | All triggers (incl. non-food) | Brown,  Ring+Messmer | Sex |
| Uasuf 2015^66^ | Retrospective case series | High | Italy | Not stated | 133 | 23 | Adults | Peach | Mueller Grade 3/4 | IgE sensitisation |
| De Schryver 2016^93^ | Retrospective case series | High | Canada | Industry and non- industry | 164 | Not stated | 2-12y  Mean 7y | All foods | Brown | Mast cell tryptase |
| Deschildre 2016^28^ | Prospective cohort | Moderate | France, Belgium, Luxemburg | Non-industry | 669 | 200 | Median 9y  (IQR 6-13)  14% >16y | Peanut | 2+ organs or anaphylaxis | Age, other atopic disease, IgE sensitisation |
| Grabenhenrich 2016^103^ | Anaphylaxis registry | Moderate | Europe | Internal | 1970  1092 food | 18 food | ≤18 y | All triggers (not just food) | Ring+Messmer Grade 3+ICU or Grade 4 | Trigger |
| Jiang 2016^104^ | Retrospective case series | High | China | Non-industry | 1501 food | 737 | 0.4-75 y  Mean 30y | All foods | Life-threatening anaphylaxis | Age, trigger, cofactors |
| Mullins 2016^107^ | Fatality case series | Moderate | Australia | Non-industry | 22 | 22 | 4-66 y  Median 28y | All foods | Fatal anaphylaxis | Age, sex, trigger, location of reaction |
| Versluis 2016^132^ | Retrospective cohort | High | Netherlands | Industry and non- industry | 496 | 258 | Mean 33 y  (sd 12.5) | All foods | Mueller grade 3/4 | Cofactors |
| Stensgaard 2017 | Cross-sectional study | High | Denmark | Not stated | 369 | N/A | Mean 15 y  (sd 8.1 y) | Peanut, hazelnut, egg, | N/A | Severity of prior reactions and impact on HRQL. |
| Chan 2017^67^ | Open FC, prospective | Low | Australia | Non-industry | 726 | 19 | Age 1-4y | Peanut, egg, sesame | Anaphylaxis (ASCIA) | IgE sensitisation |
| Abrams 2017^105^ | Open FC, retrospective | High | Canada | Internal | 104 | 20 | ≤18y  Median 5.5y | All foods | Study-defined anaphylaxis | Trigger |
| Motosue 2017^37^ | Retrospective case series | High | USA | Internal | 10464 | 591 | Adults + children | All foods | Hospital/ICU admission | Age, comorbidities |
| Nieto-Nieto 2017^36^ | Population hospital data | Moderate | Spain | Internal | 5261 | Not stated | Median 49y, IQR 43y | All triggers | ICU/mechanical ventilation | Age, comorbidities |
| Yanagida 2017^38^ | DBPCFC, retrospective | Moderate | Japan | Non-industry | 393 | 98 | Children >5y  Median 8.3y | Milk, egg, wheat, peanut | Study-defined | Age, prior history, trigger |
| Datema 2018^57^ | Prospective DBPCFC (RCT) | Moderate | Europe | Non-industry | 423  87 with FC | 116  32 FC | Adults + children | Hazelnut | Study-defined anaphylaxis | IgE sensitisation |
| Reier-Nilsen 2018^16^ | Prospective DBPCFC (RCT) | Moderate | Norway | Industry and non-industry | 96 | Not stated | 5-15 y  Median 9.7y | Peanut | EAACI 2007, Sampson | Age, sex, prior history, other atopic disease, IgE sensitisation, BAT |
| Yanagida 2018^17^ | Open FC, retrospective | Moderate | Japan | Non-industry | 979 | 334 | Children >5y  Median 8.3y | Milk, egg, wheat, peanut | NIAID | Age, prior history, trigger, IgE sensitisation |
| Worm 2018^41^ | Anaphylaxis registry | Moderate | Europe | Internal | 2588 food | 953 | Children + Adults | All foods | Ring & Messmer | Age, sex, cofactors, comorbidities |
| Pettersson 2018^15^ | Prospective DBPCFC (RCT) | Low | Netherlands | Internal | 734 | 270 | ≤18y  Median 6y | Milk, egg, peanut, hazelnut, cashew | Astier Grade 4 | Age, IgE, eliciting dose, prior history, other atopic disease |
| Kennard 2018^134^ | Retrospective case series | High | UK | Internal | 132 | 87 | Adults | WDEIA | Brown | IgE sensitisation, cofactors |
| Dua 2018^94^ | Open+blinded FC, prospective | Low | UK | Non-industry | 160 | 14 | Adults | Peanut | Ewan & Clark | Mast cell tryptase |
| Christensen 2018^135^ | Open FC, prospective | Low | Denmark | Internal | 71  46 with +ve FC | Not stated | 20-73 y  Mean, 43y | Wheat (WDEIA) | Sampson | Exercise |
| Chinthrajah 2018^40^ | Prospective DBPCFC (RCT) | Moderate | USA | Non-industry | 120 | 22 | 4-18 y  Median 11y | Peanut | Study-defined | Age, sex, other atopic disease, prior history, IgE sensitisation |
| Palosuo 2018^68^ | Prospective open+blinded FC | Low | Finland | Non-industry | 124 | 6 | Children  Median 6.3 y | Egg | Hourihane 2005 | IgE sensitisation |
| Pouessel 2018^109^ | Prospective cohort | Moderate | France | Not stated | 62 | 44 | Children | All foods | Ring & Messmer Grade 3/4 with ICU admission | Trigger |
| Arkwright 2018^19^ | Open+blinded FC, retrospective | Moderate | UK, Ireland, Australia | Non-industry | 525 | 55 | Children | Peanut | Anaphylaxis (ASCIA) | Age, eliciting dose, prior history |
| Purington 2018^39^ | DBPCFC, retrospective | Moderate | USA | Non-industry | 410 | 98 | 1-52y  Median 9y | All foods | Study-defined | Age, sex, other atopic disease, IgE sensitisation |
| Scala 2018^59^ | Retrospective case series | Moderate | Italy | Non-industry | 626 | 468 | 1-82 y  Mean 29±17y) | LTP allergy | Lower respiratory symptoms | Co-sensitisation to profilins |
| Versluis 2019^45^ | Prospective cohort | Moderate | Netherlands | Industry and non-industry | 157 | 41 | 18-70y  Mean 35y | All foods | Mueller grade 3/4 | Cofactors |
| Datema 2019^69^ | Open+blinded FC, retrospective | Moderate | Denmark | Industry and non-industry | 181 | 118 | 0.6‐27 y  Mean 6.5 y | Peanut | Sampson grade 3/4 | IgE sensitisation |
| Tejedor-Alonso 2019^139^ | Systematic review | Low-moderate | Variable | Internal | 15 studies  15,072 patients | Not stated | Not stated | All foods | Varied with study | ACE inhibitors,  beta-blockers |
| Pouessel 2019^12,43^ | Case series | Moderate | France | Industry and non-industry | 18 | 18 | 6-62y  Median 15y | All foods | Fatal anaphylaxis,  PICU admission | Age, sex, trigger, location of reaction |
| Ballmer-Weber 2019^70^ | Open+blinded FC, prospective | Moderate | Switzerland, Germany, and Spain | Incomplete declaration | 91  15 DBPCFC  46 open FC  30 anaphylaxis | 70  40 with FC | Children + Adults | Walnut | Systemic reaction | IgE sensitisation |
| Ramsey 2019^44^ | Case series -ICU data | Moderate | USA, Canada | Internal | 1989 | 19 | Children | All foods | ICU admission | Age, sex, ethnicity, asthma, trigger |
| Dua 2019^13^ | RCT | Low | UK | Non-industry | 100 | Not stated | Adults | Peanut | Adrenaline use | Co-factors |
| Francuzik 2019^144^ | Anaphylaxis registry | Moderate | Europe | Internal | 5765  1162 food | 42  9 food | Adults + children | All triggers | 3+ doses of adrenaline | Comorbidities, prior history, treatment |
| Shaker 2020^147^ | Systematic review | Low | Variable | Internal | 32 studies | Not stated | Adults + children | All triggers | Biphasic anaphylaxis | Trigger, severity of initial symptoms, treatment |
| Poirot 2020^110^ | Fatality case series | Moderate | USA | Internal | 24 | 24 | Adults + children | All foods | Fatal anaphylaxis | Age, trigger |
| Kiewiet 2020^71^ | Retrospective case series | Moderate | Sweden | Non-industry | 128 | 60 | 19-76 y  Median 51y | Alpha-gal (meat) | Study-defined | Other atopic disease, IgE sensitisation |
| Santos 2020^72,74^ | Open FC, prospective | Moderate | UK | Non-industry | 117 | 13 | 5-6 y | Peanut | CTCAE grade severe | IgE sensitisation, BAT |
| Olabarri 2020^46^ | Prospective cohort | Moderate | Spain | Non-industry | 453 episodes of anaphylaxis  396 due to food | 61 | Median 5 y  (IQR 2-9 y) | All foods | 2+ doses of adrenaline, biphasic reaction, intubation, ICU | Prior history, trigger, asthma, symptom onset |
| Kraft 2020^146^ | Anaphylaxis registry | Moderate | Europe | Internal | 9171  3343 food | 435  158 food | Adults + children | All triggers | Biphasic reaction | Comorbidities, prior history, treatment |
| Su 2020^153^ | Retrospective case series | Moderate | USA | Non-industry | 203 | 19 | Adults + children | All triggers | Poor weight gain | FPIES |
| Kaur 2021^75^ | Open FC, prospective | Moderate | Australia | Internal | 89 | 30 | Median 9 y  (IQR 6-12y) | Peanut | Study-defined | IgE sensitisation |
| Goldberg 2021^76^ | Open FC, prospective | Moderate | Israel | Internal | 120 | 60 | Median 8 y  (IQR 6-11y) | Walnut | WAO 2010 | IgE sensitisation, BAT |
| Tejedor-Alonso 2021^52^ | Systematic review | Low-moderate | Variable | Internal | 13 studies | Not stated | Not stated | All triggers | Varied with study | Respiratory disease (asthma, COPD) |
| Yonkof 2021^119^ | Open FC, retrospective | Moderate | USA | Internal | 158 | Not stated | Children | Egg, milk, nuts | NIAID | Trigger |
| Maris 2021^47^ | Anaphylaxis registry | HIgh | Europe | Internal | 1962 | 304 | ≤17 years | All foods | Ring & Messmer G3/4 | Trigger |
| Baseggio Conrado 2021^108^ | Fatality case series | Moderate | UK | Non-industry | 187 | 187 | Adults + children | All foods | Fatal anaphylaxis | Age, sex, trigger |
| Miceli Sopo 2021^154^ | Open FC (FPIES), retrospective | Moderate | Italy | Not stated | 91  48 with +ve FC | 4 | ≤10y  Mean 2y | All foods | ICON FPIES guideline | Trigger, treatment |
| Gabrielli 2021^48^ | Case registry (prospective+ retrospective) | Moderate | Canada | Non-industry | 3498  2769 food | 240 | Median 8y  (IQR 3-16y)  20% ≥16y | All triggers | EAACI 2007; admission±ICU | Age, sex, treatment, asthma |
| Gabrielli 2021^55^ | Case registry (prospective+ retrospective) | Moderate | Canada | Non-industry | 250 | 27 | Median 10y  (IQR 3-23y) | Fruit only | EAACI 2007 | Age, sex, trigger, treatment, other atopy, season |
| Lyons 2021^49^ | Prospective cohort | Moderate | Europe | Non-industry | 531  336 with probable FA | 90 | Mean 30y (sd ±13.9 y)  15% <18y | Walnut | Study-defined anaphylaxis | Age, other atopy, IgE sensitisation |
| Kraft 2021^111^ | Anaphylaxis registry | High | Europe | Internal | 1691  250 wheat | 667  153 wheat | 13+ y | Wheat | Brown | Trigger |
| Lam 2021^56^ | Population hospital data | Moderate | UK | Internal | 15,405 | N/A | Adults + children | All foods | Hospital admission | Month of admission |
| Turner 2021^18^ | Prospective DBPCFC (RCT) | Low | UK, Spain | Non-industry | 83 | 16 | Children  6-18 y, median 10 y | Cow’s Milk | Anaphylaxis (WAO 2020) | Age, sex, asthma, other atopy, IgE sensitisation |
| Błażowski 2021^51^ | Retrospective case series | Moderate | Poland | Not stated | 421 | 181 | Children  Median 3 y | All foods | Mueller 3/4 | Age, sex, asthma, other atopy, IgE sensitisation |
| Baseggio Conrado 2021^102^ | Systematic review | Low | Variable | Internal | 65 studies | Not stated | Adults + children | All triggers | Study-defined anaphylaxis | Trigger |
| Kennedy 2021^50^ | Open FC, retrospective | High | USA | Internal | 675 | 128 | ≤18 y  Medial 6 y | All foods | Study-defined | Age, asthma, trigger |
| Datema 2021^58^ | Prospective cohort | Moderate | Europe | Non-industry | 393 | 177 | Adults + children  18% ≤14y | Peanut | Study-defined | Age of onset, sex, other atopy, IgE sensitisation |

**Table S4**: Risk of bias assessment

| **Study** | **Selection**  **bias** | **External validity*** | **Case definition valid?** | **Data collection valid and systematic?** | **Recall bias** | **Internal validity**** | **Overall risk**  **of bias** |
| --- | --- | --- | --- | --- | --- | --- | --- |
| Taylor 2010**^115^** | Low | + | ++ | + | Low | + | Low |
| Pastorello 2011**^84^** | Moderate | ± | ± | ± | High | ± | High |
| Calvani 2011**^10^** | Moderate | + | + | ± | Moderate | + | Moderate |
| Huang 2012**^100^** | Moderate | ± | + | ± | High | ± | High |
| Neuman-Sunshine 2012**^27^** | Moderate | ± | + | ± | High | ± | High |
| Dang 2012**^77^** | Low | + | + | ++ | Low | + | Low |
| Eller 2012**^114^** | Moderate | + | ++ | + | Low | + | Moderate |
| Nguyen-Luu 2012**^6^** | High | ± | + | + | High | ± | High |
| Rolinck-Werninghaus 2012**^29^** | Moderate | + | ++ | ++ | Low | ++ | Moderate |
| Cianferoni 2012**^20^** | Moderate | ± | ± | ± | Moderate | ± | High |
| Vetander 2012**^53^** | Moderate | + | + | ± | Moderate | ± | Moderate |
| Eller 2013**^61^** | Moderate | + | ++ | + | Low | + | Moderate |
| Masthoff 2013**^63^** | High | ± | ± | + | Moderate | ± | High |
| van Erp 2013**^14^** | Low | + | + | + | Low | ++ | Low |
| Brown 2013**^31^** | Low | ++ | ++ | + | Low | ++ | Low |
| Libbers 2013**^124^** | Moderate | + | + | + | Low | + | Moderate |
| Klemens 2013**^62^** | Moderate | + | + | + | Low | ++ | Moderate |
| Mulla 2013**^30^** | Moderate | + | ± | + | Moderate | ± | Moderate |
| Johnson 2014**^101^** | Moderate | + | ± | ± | Moderate | + | Moderate |
| Vetander 2014**^11^** | Low | + | + | + | Moderate | + | Moderate |
| Clark 2014**^33^** | High | ± | ± | + | Moderate | ± | High |
| Jerschow 2014**^106^** | Moderate | + | ++ | + | Low | + | Low |
| Xu 2014**^32^** | Moderate | ± | ++ | + | Moderate | + | Moderate |
| Nassiri 2015**^131^** | Moderate | ± | + | ± | High | ± | High |
| Turner 2015**^34^** | Moderate | + | ++ | + | Low | + | Moderate |
| Song 2015**^35^** | Moderate | ± | + | + | Low | + | Moderate |
| Kukkonen 2015**^65^** | Moderate | + | + | + | Low | + | Moderate |
| Francuzik 2015**^42^** | Moderate | ± | + | + | Moderate | ± | Moderate |
| Uasuf 2015**^66^** | High | ± | + | ± | Low | - | High |
| De Schryver 2016**^93^** | Moderate | ± | + | ± | Moderate | ± | Moderate |
| Deschildre 2016**^28^** | Moderate | ± | + | ± | High | ± | High |
| Grabenhenrich 2016**^103^** | Moderate | ± | + | + | Moderate | ± | Moderate |
| Jiang 2016**^104^** | High | ± | + | ± | High | - | High |
| Mullins 2016**^107^** | Moderate | + | ++ | + | Low | + | Moderate |
| Versluis 2016**^132^** | Moderate | ± | ± | + | High | ± | High |
| Stensgaard 2017 | High | ± | ± | ± | High | + | High |
| Chan 201**7^67^** | Low | + | + | ++ | Low | + | Low |
| Abrams 2017**^105^** | High | ± | + | + | Moderate | + | High |
| Motosue 2017**^37^** | High | + | ± | ± | Low | ± | High |
| Nieto-Nieto 2017**^36^** | Moderate | + | ± | + | Moderate | ± | Moderate |
| Yanagida 2017**^38^** | Moderate | + | ± | + | Low | + | Moderate |
| Datema 2018**^57^** | Moderate | ± | + | + | Low | + | Moderate |
| Reier-Nilsen 2018**^16^** | Moderate | ± | + | ++ | Low | + | Moderate |
| Yanagida 2018**^17^** | Moderate | + | ± | + | Low | + | Moderate |
| Worm 2018**^41^** | Moderate | ± | + | + | Moderate | ± | Moderate |
| Pettersson 2018**^15^** | Moderate | + | + | ++ | Low | + | Low |
| Kennard 2018**^134^** | High | ± | + | ± | High | + | High |
| Dua 2018**^94^** | Moderate | + | + | + | Low | + | Low |
| Christensen 2018**^135^** | Moderate | + | + | + | Low | + | Low |
| Chinthrajah 2018**^40^** | Moderate | ± | + | + | Low | + | Moderate |
| Palosuo 2018**^68^** | Moderate | + | + | ++ | Low | + | Low |
| Pouessel 2018**^109^** | Moderate | ± | + | ± | Moderate | ± | Moderate |
| Arkwright 2018**^19^** | Moderate | ± | + | ± | Moderate | ± | Moderate |
| Purington 2018**^39^** | Moderate | ± | + | + | Moderate | ± | Moderate |
| Scala 2018**^59^** | Moderate | + | + | + | Moderate | + | Moderate |
| Versluis 2019**^45^** | Moderate | ± | + | + | Low | + | Moderate |
| Datema 2019**^69^** | Moderate | + | ++ | + | Low | + | Moderate |
| Tejedor-Alonso 2019**^139^** | N/A | N/A | + | ± | N/A | + | Low-moderate |
| Pouessel 2019**^12,43^** | Moderate | + | ++ | + | Low | + | Moderate |
| Ballmer-Weber 2019**^70^** | Moderate | + | ± | + | Moderate | + | Moderate |
| Ramsey 2019**^44^** | Moderate | + | + | + | Low | + | Moderate |
| Dua 2019**^13^** | Moderate | + | + | ++ | Low | ++ | Low |
| Francuzik 2019**^144^** | Moderate | ± | + | + | Moderate | ± | Moderate |
| Shaker 2020**^147^** | N/A | + | + | + | N/A | + | Low |
| Poirot 2020**^110^** | Moderate | + | + | ± | Low | + | Moderate |
| Kiewiet 2020**^71^** | Moderate | ± | + | + | Moderate | ± | Moderate |
| Santos 2020**^72,74^** | Moderate | + | ± | + | Low | ± | Moderate |
| Olabarri 2020**^46^** | Moderate | + | + | ± | Low | + | Moderate |
| Kraft 2020**^146^** | Moderate | ± | + | + | Moderate | ± | Moderate |
| Su 2020**^153^** | Moderate | + | ± | ± | Low | + | Moderate |
| Kaur 2021**^75^** | Moderate | + | + | + | Low | + | Moderate |
| Goldberg 2021**^76^** | Moderate | ± | + | + | Low | + | Moderate |
| Tejedor-Alonso 2021**^52^** | N/A | N/A | + | ± | N/A | + | Low-moderate |
| Yonkof 2021**^119^** | High | ± | + | + | Low | + | Moderate |
| Maris 2021**^47^** | Moderate | ± | ± | ± | Moderate | ± | High |
| Baseggio Conrado 2021**^108^** | Moderate | + | ++ | + | Low | + | Moderate |
| Miceli Sopo 2021**^154^** | Moderate | + | + | + | Moderate | + | Moderate |
| Gabrielli 2021**^48^** | Moderate | ++ | + | + | Low | + | Moderate |
| Gabrielli 2021**^55^** | Moderate | ++ | + | + | Low | + | Moderate |
| Lyons 2021**^49^** | Moderate | ± | + | ++ | Moderate | ++ | Moderate |
| Kraft 2021**^111^** | Moderate | ± | ± | ± | Moderate | ± | High |
| Lam 2021**^56^** | Moderate | + | ± | + | Moderate | + | Moderate |
| Turner 2021**^18^** | Moderate | + | + | ++ | Low | ++ | Low |
| Błażowski 2021**^51^** | Moderate | + | + | ± | Moderate | + | Moderate |
| Baseggio Conrado 2021**^102^** | Moderate | ++ | ± | ± | Low | + | Moderate |
| Kennedy 2021**^50^** | High | + | + | ± | Moderate | ± | High |
| Datema 2021**^58^** | Moderate | ± | ± | + | Moderate | + | Moderate |

*External validity assesses for whether selection bias impacts on whether the study data are generalizable to the overall food-allergic population, and described as ++ (all or most of the criteria have been fulfilled, and where not the conclusions are very unlikely to alter), + (some criteria have been fulfilled, and where not fulfilled or adequately described, the conclusions are unlikely to alter), ± (few or no checklist criteria fulfilled)

**Internal validity reflects the degree of systematic data collection and how this data was sourced (e.g. direct from patients, contemporaneous medical notes, historical case notes)

**Table S5**: Excluded studies

| Study | Reason not eligible |
| --- | --- |
| Ballini et al. Frequency of positive oral food challenges and their outcomes in the allergy unit of a tertiary-care pediatric hospital. Allergol Immunopathol (Madr). 2021;49(3):120-130. | Only 14 positive challenges FPIES challenges reported. No robust analysis of severity for OFC for IgE-mediated food allergy. |
| Błażowski et al. Food allergy endotype with high risk of severe anaphylaxis in children-Monosensitization to cashew 2S albumin Ana o 3. Allergy. 2019 Oct;74(10):1945-1955. | Unclear how many individuals with anaphylaxis included. “Severe” anaphylaxis cohort included 77 children. Overlap with Błażowski et al 2021. |
| Buka et al. Anaphylaxis and ethnicity: higher incidence in British South Asians. Allergy. 2015;70(12):1580-7. | Only 38 anaphylaxis reactions to food included. |
| Hompes et al. Elicitors and co-factors in food-induced anaphylaxis in adults. Clin Transl Allergy. 2013 Nov 21;3(1):38. | <50 food-allergic individuals with positive FC included. |
| Kim et al. Clinical Manifestations and Risk Factors of Anaphylaxis in Pollen-Food Allergy Syndrome. Yonsei Med J. 2019;60:960-968. | No FC reported, and <500 participants. |
| Klingebiel et al. Pru p 7 sensitization is a predominant cause of severe, cypress pollen-associated peach allergy. Clin Exp Allergy. 2019 Apr;49(4):526-536. | Only 78 patients with prior anaphylaxis included (<100). |
| Kotaniemi-Syrjänen et al. Likelihood of Immediate Food Challenge Reactions Varies by Age, History, Allergens, and Levels of Sensitization. Pediatric Allergy, Immunology, and Pulmonology 2017.45-52. | No analyses in terms of severity following FC. |
| Lee et al. Antihypertensive medication use is associated with increased organ system involvement and hospitalization in emergency department patients with anaphylaxis. JACI 2013;131(4):1103-8. | Only 82 (<100) food-allergic individuals with anaphylaxis included. |
| Masthoff et al. Diagnostic value of hazelnut allergy tests including rCor a 1 spiking in double-blind challenged children. Allergy. 2012;67:521-7. | Only 32 objective reactions to hazelnut included. |
| Sahiner et al. Serum basal tryptase may be a good marker for predicting the risk of anaphylaxis in children with food allergy. Allergy. 2014;69(2):265-8. | <100 food-allergic individuals with anaphylaxis included. |
| Sala-Cunill et al. Usefulness and limitations of sequential serum tryptase for the diagnosis of anaphylaxis in 102 patients. Int Arch Allergy Immunol. 2013;160(2):192-9. | Only 35 reactions to food included. |
| Sánchez-Ruano et al. Clinical utility of microarray B-cell epitope mapping in food allergies: A systematic review. Pediatr Allergy Immunol. 2020;31(2):175-185. | No analyses relating to severity reported. |
| Santos et al. Distinct parameters of the basophil activation test reflect the severity and threshold of allergic reactions to peanut. JACI 2015;135(1):179-86. | Overlap with Santos et al 2020. |
| Srivastava et al. Systemic reactions and anaphylaxis with an acute serum tryptase ≥14 μg/L: retrospective characterisation of aetiology, severity and adherence to NICE guidelines for serial tryptase measurements and specialist referral. J Clin Pathol. 2014;67(7):614-9. | Only 10 reactions to food included. |
| Ta et al. Use of Specific IgE and Skin Prick Test to Determine Clinical Reaction Severity. Br J Med Med Res. 2011;1(4):410-429. | N=24 only. |
| Wang et al. Food Protein-Induced Enterocolitis Syndrome Food Challenges: Experience from a Large Referral Center. JACI Pract. 2019 Feb;7(2):444-450. | Only 30 challenges positive (<50), with most FC undertaken to demonstrate resolution. Analysis of risk factors for historical severity not possible. |
| Yoneyama et al. Probability curves for predicting symptom severity during oral food challenge with milk. Ann Allergy Asthma Immunol. 2015 Sep;115(3):251-3. | Did not distinguish between systemic reactions and anaphylaxis (combined all Sampson Grade 3+ reactions together). |

**Table S6**: Evidence for asthma as a possible risk factor in severe food allergic reactions

| **Study** | **Population** | **Outcome** | **Was asthma a risk factor?** |
| --- | --- | --- | --- |
| Calvani 2011^10^ | Food-allergic children | Sampson grade 4/5 reaction | History of asthma associated with wheeze during anaphylaxis (OR 2.2, 95% CI 1.1–4.5) and Grade 4/5 anaphylaxis (aOR) 7.1, 95% CI 2.5–20.2) |
| Neuman-Sunshine 2012^27^ | Peanut-allergic children (clinic population) | Wheeze during accidental reactions | Asthma *not* associated with respiratory symptoms (OR 1.29, 95% CI 0.83–2.01) |
| Rolinck-Werninghaus 20122^29^ | Food challenge (72% DBPCFC) to milk, egg, wheat, soya in children | Grade 4/5 reaction  (adapted from Sampson) | Asthma *not* associated with more severe anaphylaxis at challenge |
| van Erp 2013^14^ | DBPCFC to peanut (children) | Wheeze at challenge | Asthma with prescribed therapy *not* associated with respiratory symptoms  (OR 1.40, 95% CI 0.60-3.25) |
| Mulla 2013^30^ | State health database  (children+adults) | ICU/mechanical ventilation (all cause anaphylaxis) | Asthma *not* associated with ICU admission (aOR 1.04, 95% CI 0.82-1.33)  Asthma associated with increased risk of ventilation (aOR 2.45, 95% CI 1.81 to 3.33) |
| Brown 2013^31^ | Food-induced anaphylaxis (Emergency Dept., all ages) | Brown Grade 3 reaction | Asthma *not* associated with more severe reactions (any trigger: OR 0.69, 95% CI 0.41-1.15; food only: OR 1.07, 95% CI 0.40-2.84) |
| Xu 2014^32^ | Coroner’s database  (children+adults) | Fatal anaphylaxis  (all triggers) | Asthma status determined in 28/92 fatalities (30%): 26 had asthma, 8/26 poorly controlled. High risk of bias as 70% fatalities had unknown asthma status |
| Clark 2014^33^ | State health database  (children+adults) | Admission due to anaphylaxis (food only) | Weak association between asthma diagnosis and hospital admission (OR 4.77, 95% CI 0.79-28.68), but high risk of bias as only 0.3% of cases hospitalised |
| Turner 2015^34^ | Fatal anaphylaxis register  (children+adults) | Fatal anaphylaxis to food | 97/124 (78%) of 124 fatal cases were in patients with asthma. Age distribution of fatal asthma is different to that seen for fatal food-induced anaphylaxis |
| Song 2015^35^ | DBPCFC in FAHF study (teenagers+adults) | Sampson grade 4/5 reaction | Asthma *not* associated with Grade 4 reactions at DBPCFC (OR 1.57, 95% CI 0.43-5.83) |
| Deschildre 2016^28^ | Children and adults with food allergy | Anaphylaxis as historical reaction | Weak association between diagnosis of asthma and anaphylaxis (OR 1.46, 95% CI 1.04-2.05) |
| Nieto-Nieto 2017^36^ | State health database  (children+adults) | Mechanical ventilation due to anaphylaxis (all cause) | Asthma associated with increased risk of ventilation (aOR 2.23, 95% CI 1.55-3.22) |
| Motosue 2017^37^ | Insurance database  (all ages, all cause) | Admission to hospital or ICU/intubation | Asthma *not* associated with: severity (aOR 0.93, 95% CI 0.89-0.97), ICU admission (aOR 0.88, 95% CI 0.82-0.93) or intubation (aOR 0.84, 95% CI, 0.75-0.95) |
| Yanagida 2017^38^ | DBPCFC to milk, egg, wheat, peanut (children) | Severe anaphylaxis at DBPCFC (as per JSA) | Asthma *not* associated with more severe anaphylaxis at DBPCFC  (OR 0.32, 95% CI 0.13-0.54) |
| Pettersson 2018^15^ | DBPCFC to milk, egg, peanut, cashew, hazelnut in children | Astier grade 4 reaction at DBPCFC | In a prediction model, asthma was *not* a significant predictor for the severity of reaction at challenge or historical accidental reaction. Asthma *not* associated with Grade 4 reactions at DBPCFC (OR 0.61, 95% CI 0.45-0.83). |
| Purington 2018^39^ | DBPCFC to milk, egg, peanut, cashew, hazelnut (children+adults) | Any organ-specific symptom at grade 3 using Bock classification, at DBPCFC | History of asthma was associated with a higher risk of more severe symptoms (not specifically lower respiratory symptoms) at challenge (hazard ratio 2.37, 95% CI 1.36 to 4.13). Asthma status missing in 10%. |
| Chinthrajah 2018^40^ | Peanut-DBPCFC in POISED study (children+adults) | Symptom severity  (study-defined) | Asthma *not* associated with more severe symptoms at DBPCFC  (OR 2.61, 95% CI 0.82-8.32) |
| Reier-Nilsen 2018^16^ | Peanut-DBPCFC in TAKE-AWAY study (children) | Sampson grade 4/5 reaction | Asthma *not* associated with severity of symptoms at DBPCFC |
| Yanagida 2018^17^ | Open FC to milk, egg, wheat, peanut (children) | Severe anaphylaxis at FC (as per JSA) | Asthma *not* associated with more severe anaphylaxis at open FC  (OR 1.06, 95% CI 0.76-1.48) |
| Worm 2018^41^  Sub-analysis in Francuzik 2019^42^ | European anaphylaxis registry (children+adults) | Hypoxemia, hypotension, collapse (all cause anaphylaxis)  Refractory anaphylaxis (3+ adrenaline doses needed) | Asthma *not* associated with more severe anaphylaxis (aOR 0.75, 95% CI 0.61 -0.88). 36% of cases due to food, no sub-analysis of impact on severity reported for food.  Asthma associated with refractory anaphylaxis (aOR 2.91, 95% CI 1.48-5.71). Food accounted for 24% of cases. Only 9 cases of refractory anaphylaxis due to food. |
| Pouessel 2019^43^ | Fatality register  (children+adults) | Fatal anaphylaxis to food | 11/18 (61%) had a diagnosis of asthma, none documented to be on maintenance treatment for asthma |
| Ramsey 2019^44^ | ICU database (children) | Intubation due to (all-cause) anaphylaxis | Asthma diagnosis associated with increased likelihood of intubation (OR 1.46, 95% CI 1.14-1.86). No impact on mortality. |
| Versluis 2019^45^ | Food-allergic adults | Ewan & Clark grade 3-5 reaction | Asthma exacerbation *not* associated with more severe reactions  (OR 0.90, 95% CI 0.29-2.8) |
| Dua 2019^13^ | DBPCFC in peanut-allergic adults | Anaphylaxis (WAO 2022) | Asthma *not* associated with anaphylaxis at DBPCFC (OR 1.31, 95% CI 0.55-3.08) |
| Olabarri 2020^46^ | Acute food-anaphylaxis in children (Emergency Dept) | Severe reaction (2+adrenaline or biphasic reaction or ICU) | History of asthma associated with more severe anaphylaxis (aOR 2.70, 95% CI 1.43-5.11). 87% reactions due to food |
| Maris 2021^47^ | Peanut-allergic children | Respiratory symptoms | No difference in frequency of respiratory symptoms in those with/without asthma |
| Gabrielli 2021^48^ | Accidental reactions to food in adults+children | i) Hospital/ICU admission  ii) ≥2 doses of adrenaline | History of asthma *not* associated with hospital/ICU admission (aOR 1.11, 95% 0.63-1.96) or need for multiple doses of adrenaline (aOR 1.24, 95% 0.82-1.87) |
| Lyons 2021^49^ | Accidental reactions to walnut in adults+children | Anaphylaxis (study defined) | History of asthma *not* associated with more severe reactions (OR 0.88, 95% CI 0.24-4.14); but excluded from meta-analysis as 97% of cohort had asthma (possible bias) |
| Turner 2021^18^ | DBPCFC to cow’s milk | Anaphylaxis (WAO 2022) | Asthma *not* associated with anaphylaxis at DBPCFC (OR 0.82, 95% CI 0.27-2.46) |
| Kennedy 2021^50^ | Children under-going open FC to food. | Symptom severity (study-defined) | Asthma associated with more severe symptoms (not specifically lower respiratory symptoms) in a regression model. Not associated with use of adrenaline. |
| Błażowski 2021^51^ | Accidental reactions (children) | Muller grade 3/4 reaction | Asthma not associated with increased severity (OR 0.74, 95% CI 0.50-1.08). |
| Tejedor-Alonso 2021^52^ | Systematic Review | Severity of anaphylaxis | Asthma associated with increased anaphylaxis severity (OR 1.9, 95% CI 1.3-2.8), but high risk of bias. |

**Figure S1:** Sensitivity analysis of studies reporting impact of asthma on severity of allergic reactions in studies reporting food as a trigger, limited to studies reporting unadjusted odds ratios only.


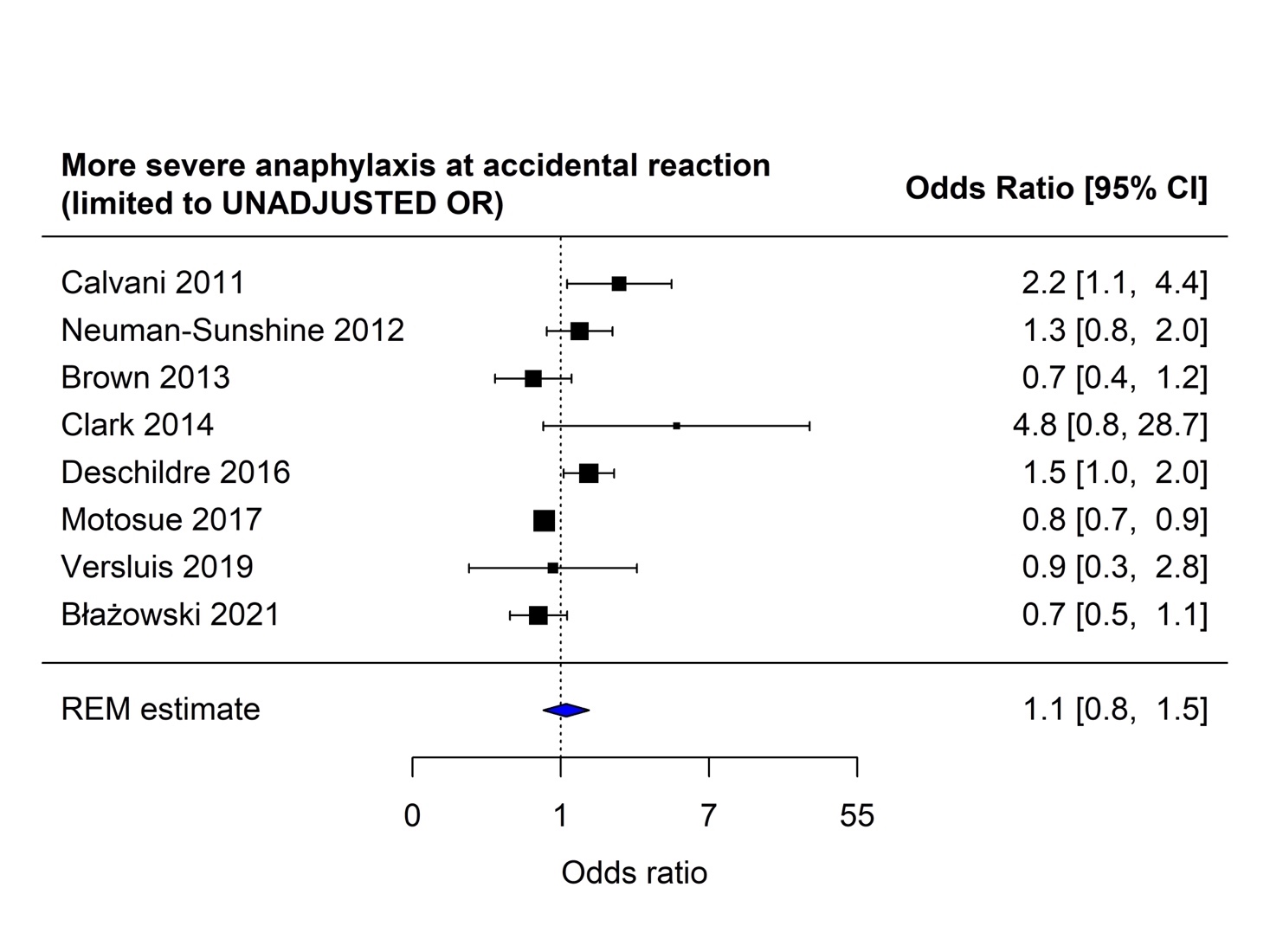


**Figure S2**: Funnel plot of studies reporting outcomes related to “More severe anaphylaxis at accidental reaction”


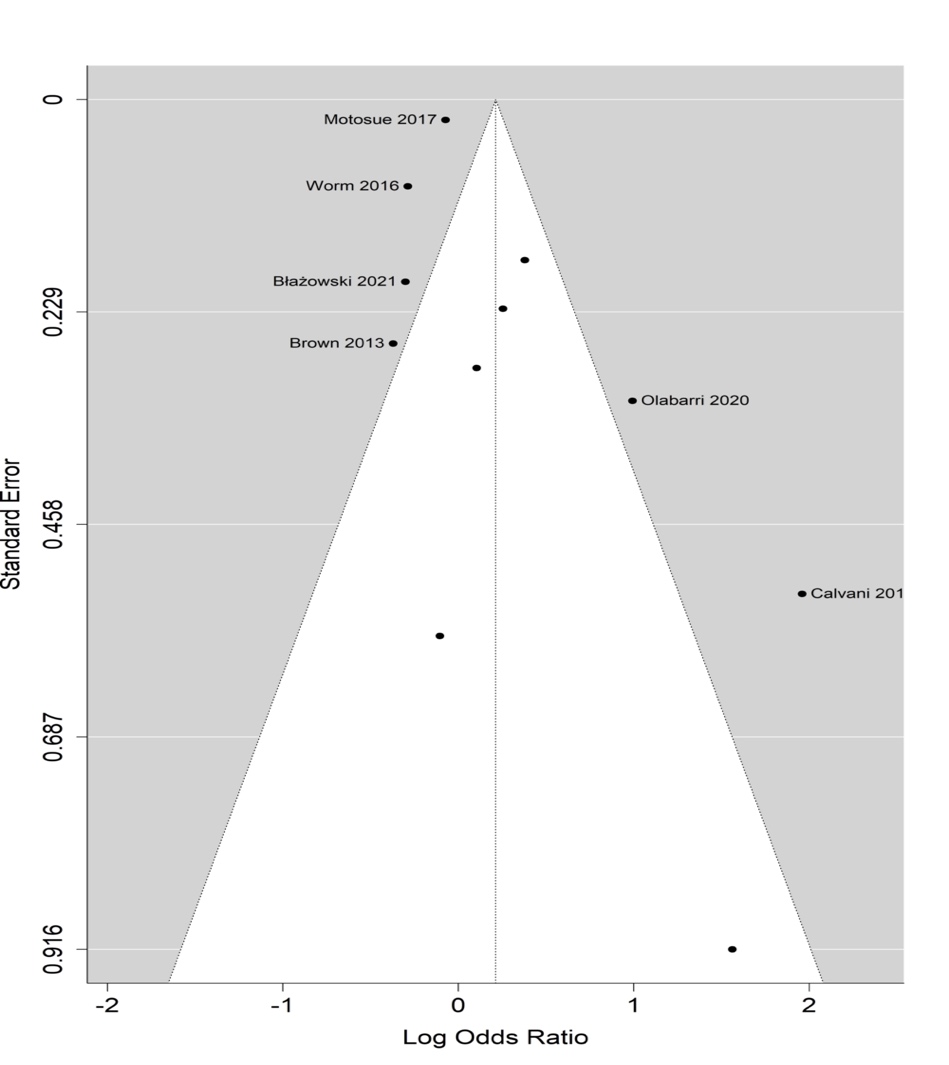


**Figure S3**: Funnel Plot of studies reporting outcomes related to “More severe anaphylaxis at food challenge”


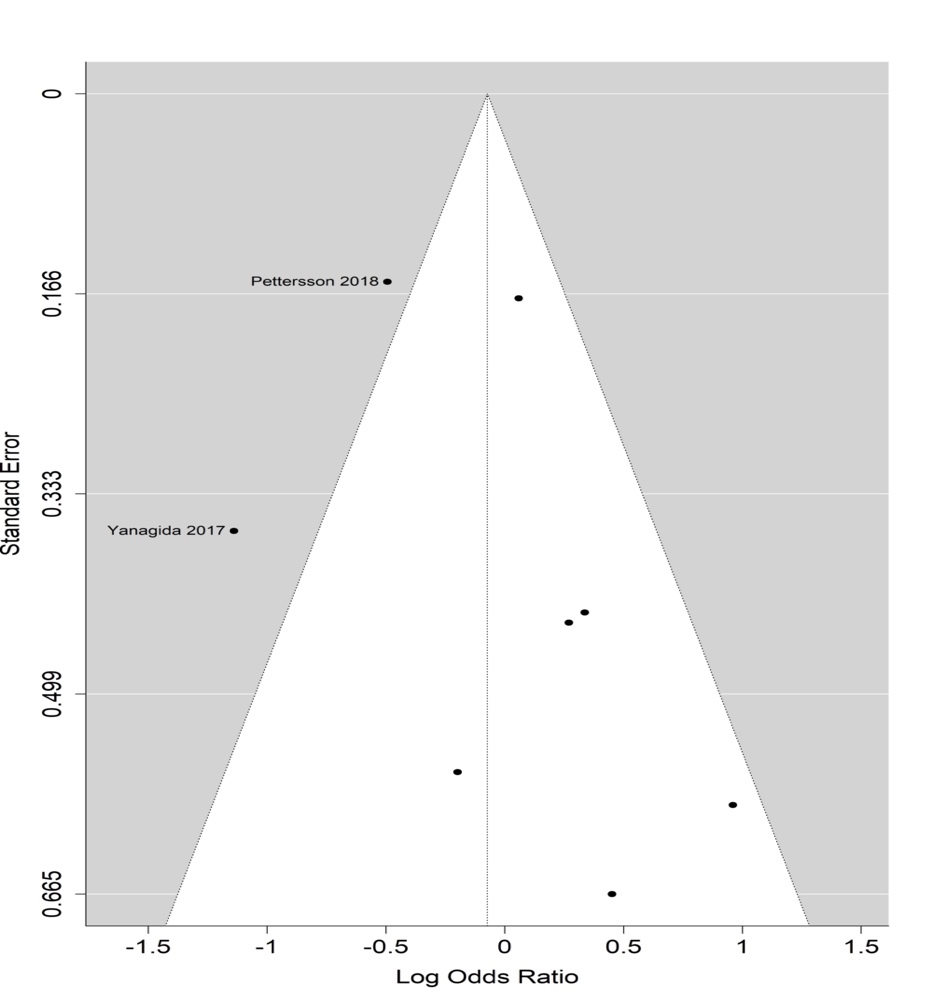


**Figure S4**: Impact of including non-allergic patients as non-severe reactors evaluating the diagnostic utility of different biomarkers to predicting the occurrence of anaphylaxis and severe reactions to peanut in the LEAP study cohort.^72-74^ Receiver-operating characteristic curves for (**A**) those with anaphylaxis and (**B**) those with severe reactions (Common Terminology Criteria for Adverse Events (CTCAE) Grade 3 reaction) compared to non-severe group. AUC, Area under the ROC curve with 95% confidence intervals. The perfect test will have an AUC of 1.0, AUC >0.8 is considered to represent excellent discrimination.


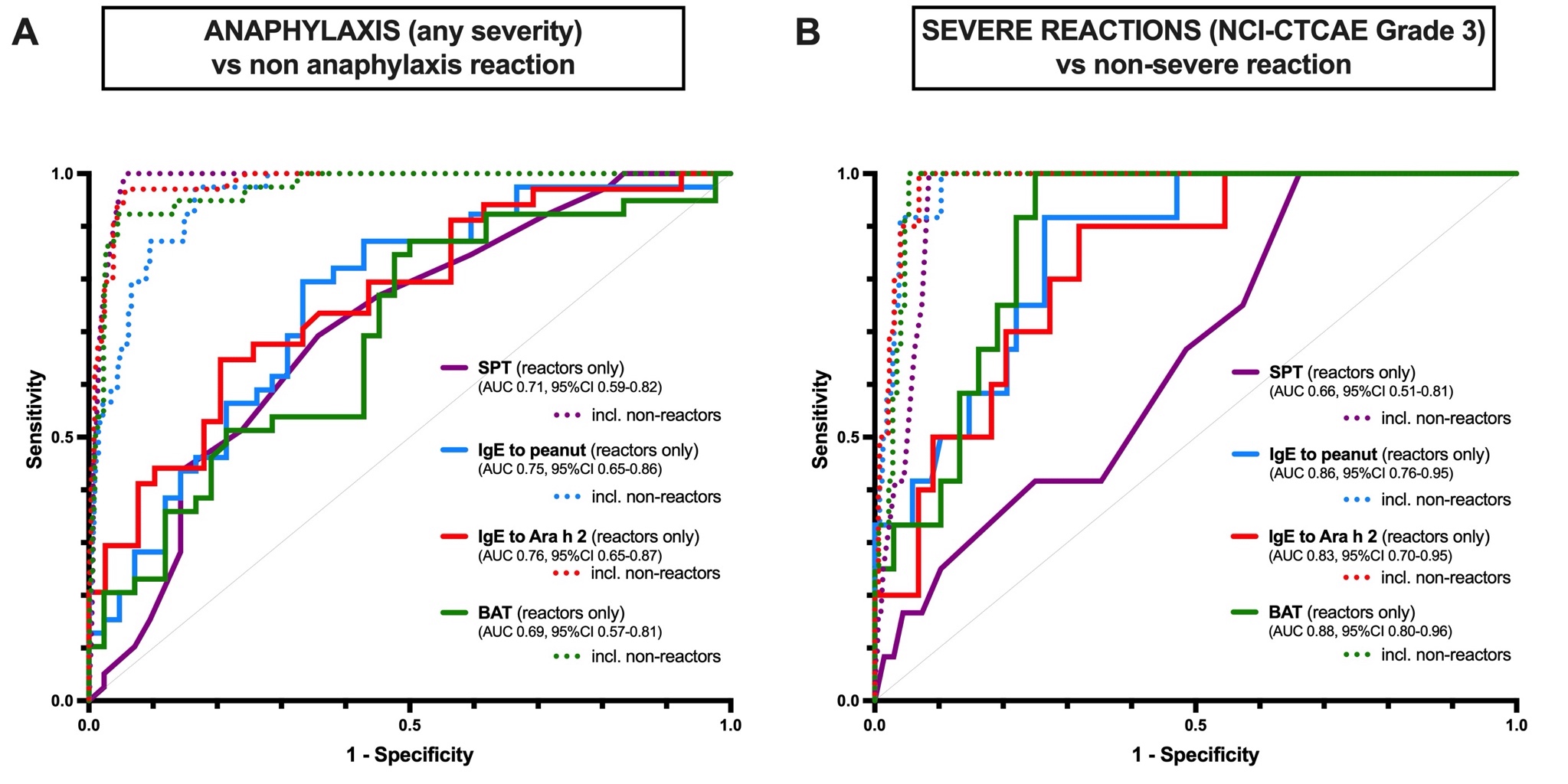

Supplement: Supplementary file 1 — Supplementary Material [file ALL-77-2634-s001.docx]
